# Duplications and functional divergence of ADP-glucose pyrophosphorylase genes in plants

**DOI:** 10.1186/1471-2148-8-232

**Published:** 2008-08-12

**Authors:** Nikolaos Georgelis, Edward L Braun, L Curtis Hannah

**Affiliations:** 1 Program in Plant Molecular and Cellular Biology and Horticultural Sciences, University of Florida, Gainesville, Florida 32610-0245, USA; 2 Department of Zoology, University of Florida, Gainesville, Florida 32611-8525, USA

## Abstract

**Background:**

ADP-glucose pyrophosphorylase (AGPase), which catalyses a rate limiting step in starch synthesis, is a heterotetramer comprised of two identical large and two identical small subunits in plants. Although the large and small subunits are equally sensitive to activity-altering amino acid changes when expressed in a bacterial system, the overall rate of non-synonymous evolution is ~2.7-fold greater for the large subunit than for the small subunit. Herein, we examine the basis for their different rates of evolution, the number of duplications in both large and small subunit genes and document changes in the patterns of AGPase evolution over time.

**Results:**

We found that the first duplication in the AGPase large subunit family occurred early in the history of land plants, while the earliest small subunit duplication occurred after the divergence of monocots and eudicots. The large subunit also had a larger number of gene duplications than did the small subunit. The ancient duplications in the large subunit family raise concern about the saturation of synonymous substitutions, but estimates of the absolute rate of AGPase evolution were highly correlated with estimates of ω (the non-synonymous to synonymous rate ratio). Both subunits showed evidence for positive selection and relaxation of purifying selection after duplication, but these phenomena could not explain the different evolutionary rates of the two subunits. Instead, evolutionary constraints appear to be permanently relaxed for the large subunit relative to the small subunit. Both subunits exhibit branch-specific patterns of rate variation among sites.

**Conclusion:**

These analyses indicate that the higher evolutionary rate of the plant AGPase large subunit reflects permanent relaxation of constraints relative to the small subunit and they show that the large subunit genes have undergone more gene duplications than small subunit genes. Candidate sites potentially responsible for functional divergence within each of the AGPase subunits were investigated by examining branch-specific patterns of rate variation. We discuss the phenotypes of mutants that alter some candidate sites and strategies for examining candidate sites of presently unknown function.

## Background

**Figure 1:**
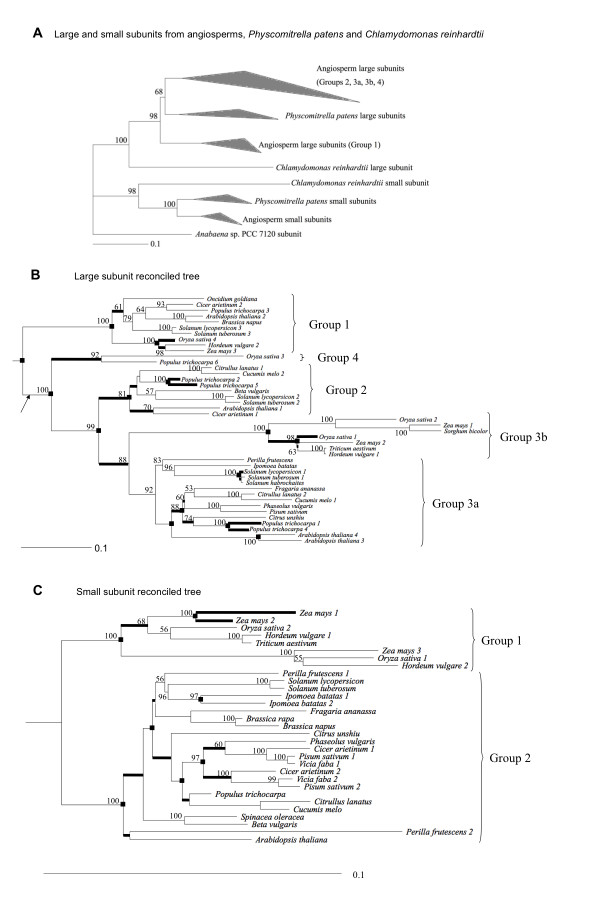
**Reconciled large and small subunit trees**. A) Amino acid tree of the large and small subunits from angiosperms, *Physcomitrella patens *and *Chlamydomonas reinhardtii*. The topology of the tree was determined by ML using aligned amino acid sequences using PhyML. Branch lengths reflect numbers of amino acid substitutions per site. The branches within groups have been replaced by the grey triangles. ML bootstrap values are indicated above branches, and the bar shows the number of amino acid substitutions per site. B) Angiosperm large subunit reconciled tree. C) Angiosperm small subunit reconciled tree. The topology of the trees shown in B) and C) was determined by ML using aligned cDNA sequences using GARLI. Branch lengths reflect numbers of amino acid substitutions per site as estimated by AAML and the scale bar shows the number of amino acid substitutions per site. ML bootstrap values > 50% are indicated above branches. Reconciled tree analyses (using the gene trees shown and the species tree shown in Additional file 1) were conducted using GENETREE. Black boxes at nodes indicate duplication events. The arrow in B) indicates the divergence of *Physcomitrella patens *from angiosperms. The trees in B) and C) were rooted with the AGPase large and small subunit from *Chlamydomonas reinhardtii *respectively. Thicker lines indicate branches that follow duplication events and have K_S _< 0.1 (based upon ML estimates of synonymous branch lengths).

ADP-glucose pyrophosphorylase (AGPase; EC 2.7.7.27) catalyses a rate-limiting step in starch synthesis, the formation of ADP-glucose from glucose-1-P and ATP. ADP-glucose is the predominant, if not sole, precursor for starch synthesis. While AGPase is a homotetramer in bacteria (including cyanobacteria), it is a heterotetramer in angiosperms and green algae. This heterotetramer comprises two identical large and two identical small subunits. They exhibit a high degree of identity to each other and to the cyanobacterial AGPase, pointing to an origin by gene duplication early in the evolution of plants and green algae (Figure 1A) (Additional file 1) [1,2]. The two subunits have complementary rather than redundant functions, and knockout mutations in either abolish more than 90% of AGPase activity in some experimental systems [3].

Although both subunits are necessary for full AGPase activity, the angiosperm small subunit appears more conserved than the large subunit throughout its sequence (small subunits exhibit an average of 91.3% amino acid identity while large subunits an average of 70.8% identity) [2]. However, mean percent identities might be misleading since the genes encoding both subunit genes of AGPase underwent a number of duplications after the initial duplication that generated the two subunits. The potential confusion due to the comparison of paralogs rather than orthologs can be overcome by methods that incorporate phylogeny, such as the use of maximum likelihood (ML) to estimate ω (the ratio of non-synonymous substitutions per non-synonymous site [K_A_] to synonymous substitutions per synonymous site [K_S_]). The ML estimate of ω for the large subunit is ~2.7-fold greater than the estimate for the small subunit [2], suggesting a higher rate of amino acid replacement for the large subunit.

Although ω provides a convenient and commonly used method to examine evolutionary constraints, it has typically been used to examine sequences that have diverged relatively recently. The rate of synonymous evolution is relatively high in plant nuclear genes [4-7] and estimates of K_S _appear saturated in analyses of some angiosperm gene families, even for relatively shallow evolutionary divergences [8]. Hence, the accuracy of ω estimates for ancient divergences is unclear. Another potential problem for the use of ω is the assumption that mutations at synonymous sites are neutral. It has been suggested that synonymous sites are subject to both positive and purifying selection [9-12]. The action of selection on synonymous sites may explain why adding among-sites rate variation for synonymous sites to models of codon evolution improves their fit to empirical data [13,14]. Saturation and among-sites rate variation both have the potential to cause K_S _to be underestimated (and ω to be overestimated since ω = K_A_/K_S_); biased estimates of ω will lead to incorrect inferences regarding evolutionary constraints on the proteins being analyzed. Finally, ω cannot detect changes in the evolutionary rate when rates of synonymous and non-synonymous substitution increase or decrease simultaneously [15].

The almost 3-fold difference in evolutionary rates for the AGPase subunits is a paradox because random mutagenesis revealed that maize endosperm AGPase subunits expressed in bacteria are equally susceptible to activity-altering amino acid changes [2]. Georgelis et al. [2] proposed that the difference in evolutionary rates between AGPase subunits reflected, at least in part, the differences between the subunits in their tissue-expression patterns and the fact that the small subunit has to interact with multiple large subunits in plants. Here, we establish the pattern and timing of duplications in the AGPase gene family and estimate absolute rates of AGPase sequence evolution. Functional divergence has been observed among AGPase subunits based on biochemical criteria [16-20]. One of our primary goals was to identify candidate sites for functional divergence. We identify specific AGPase sites apparently subject to either positive selection or branch-specific patterns of rate variation (types-I and -II divergence as defined by Gu [21,22]).

## Results

### Patterns of AGPase gene duplication

It is well known that genes can have three possible fates after duplication [23-27]: (1) nonfunctionalization, in which one duplicate is lost, (2) subfunctionalization, in which the functions of the original single-copy gene are partitioned between the duplicates, and (3) neofunctionalization, in which one duplicate gains a novel function. The latter two processes can result in paralogs that persist for a substantial length of time (although a few exceptions have been proposed, such as pseudogene resurrection [27]). Throughout this work, the term duplication will be limited to the description of the latter two processes. Although gene loss can be as important as duplication for shaping genomes [28], we have avoided making major conclusions based on gene loss since many organisms included in these analyses lack complete genome sequences.

Inclusion of AGPase sequences from the moss *Physcomitrella patens *[29], which has 7 large subunit and 4 small subunit genes, placed a major constraint on the earliest divergence within the large subunit family since the moss sequences were intermixed with angiosperm sequences. This indicates that the earliest duplication in the large subunit family occurred prior to the divergence of angiosperms and mosses, more than 400 million years (MY) ago [30]. Since the rate of synonymous evolution in angiosperms varies from ~2 × 10^-9 ^to ~10 × 10^-9 ^synonymous substitutions per synonymous site per year [4-7], values of K_S _in excess of 2 are expected for some comparisons, which may make estimates of K_S _problematic [31].

Since many divergence times for plants can be constrained to reasonable ranges, it should be possible to estimate absolute rates of AGPase subunit amino acid evolution and establish whether they correlate with estimates of ω. However, this requires differentiating between speciation and gene duplication events in AGPase phylogeny. Gene family phylogenies reflect both speciation and duplication events, and these events can be distinguished by reconciled tree analyses if the gene and species trees are known. Gene tree parsimony [32] is the most commonly used reconciled tree method, and the only approach practical for even moderately sized phylogenies at this time. Reconciling the AGPase gene trees with the best available estimate of the land plant species tree (Additional file 2) revealed 11–14 large subunit duplications (Figure 1B) and 5–7 small subunit duplications (Figure 1C). It may be appropriate to view the lower estimates, which are based upon well-supported nodes, as the primary results since they are based on modified versions of the gene trees (Additional file 3A, B) in which the topology was rearranged near poorly supported nodes to increase congruence with the species tree (Methods). In contrast, the higher estimates were based only on reconciling the optimal estimates of the gene trees (Figure 1B, C) with the species tree. Regardless, both analyses indicate that the large subunit genes underwent a larger number of duplications than the small subunit genes.

AGPase large subunits have narrower tissue-specificity than small subunits [33-38], and the large subunit phylogeny appears more complex (with four major clades some of which include both monocots and eudicots; Figure 1B) than the small subunit phylogeny (Figure 1C). Large subunit group 1 genes are predominantly expressed in leaves, group 2 genes are expressed both in source and sink tissues, group 3 genes are expressed sink tissues (these genes are subdivided into group 3a in eudicots and group 3b in monocots), and group 4 corresponds to a clade of two sequences that have not been characterized yet in terms of function and expression patterns (Figure 1B). Some of the major large subunit clades arose prior to the divergence of monocots and eudicots, and the optimal placement of the AGPase large subunit sequences from *Physcomitrella *suggests that the first duplication in the large subunit happened around 400 MY ago (Figure 1A). In contrast, there is no evidence that angiosperm small subunits underwent a duplication prior to the divergence of monocots and eudicots, and we have divided them into a monocot clade (group 1) genes and a eudicot clade (group 2). These results emphasize that the large subunit underwent a larger number of duplications than did the small subunit and that only large subunit duplications began before the divergence of monocots and eudicots.

### Absolute rates of AGPase evolution

**Figure 2:**
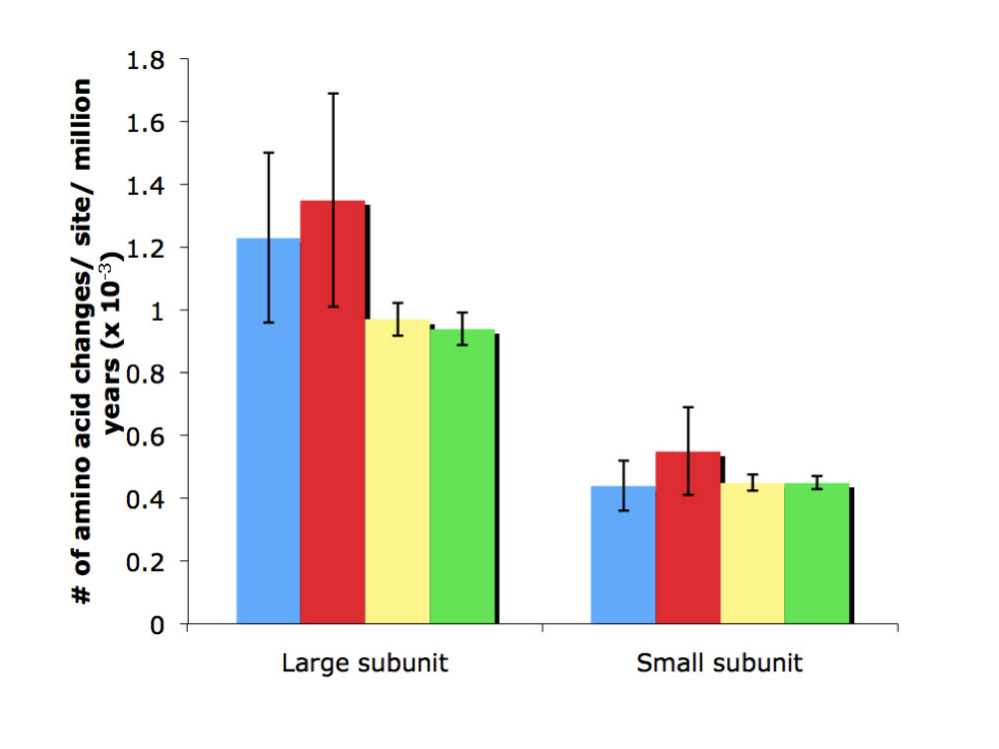
**Absolute rate of evolution of the large and the small subunit of AGPase from angiosperms (measured in aass MY^-1^)**. The blue bars indicate the average rates (amino acid substitutions per site per million years; aass MY^-1^) estimated from the most recent dated speciation events to present sequences of the trees shown in Figure 1B and 1C. The red bars indicate the average aass MY^-1 ^estimated from the most recent dated speciation events to present sequences of the trees shown in Additional file 3A and 2B. The yellow bars indicate the average aass MY^-1 ^estimated from all branches in Figure 3A and 3B. The green bars indicate the average aass MY^-1 ^estimated from all branches in Additional file 4A and 3B. The error bars indicate 2× standard error.

Absolute rates of amino acid evolution for AGPase subunits were estimated by examining terminal branch lengths for divergences that reflect speciation events with known divergence times (these divergence times are presented in Additional file 2). This approach is called the tip procedure since it involves only terminal branches (Methods), and it revealed that the average rate of evolution for the large subunit was 2.7-fold faster than that of the small subunit (Figure 2). This rate difference was both congruent with the difference in ML estimates of ω [2] and highly significant (*P *= 0.0006 by Student's unpaired *t*-test). Our conclusions were unchanged if we limited consideration to strongly supported duplication events (those retained when bootstrap support was considered; see Additional file 3).

**Figure 3:**
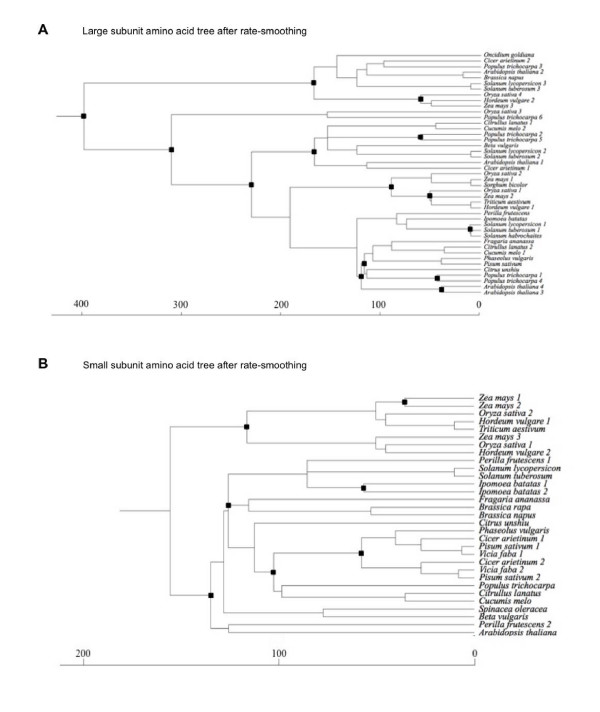
**Phylogenetic trees of the large and the small subunits from angiosperms after rate-smoothing**. The trees in A) and B) are the trees shown in Figure 1B and 1C respectively after rate-smoothing. Rate-smoothing was done by using penalized likelihood as implemented in the r8s software [79]. Black boxes indicate duplication events.

Estimates of the absolute rate of amino acid substitution for AGPase subunits obtained by the penalized likelihood (PL) method (Figure 3) were very similar to those obtained using the tip procedure (Figure 2). Using gene trees in which poorly supported nodes were rearranged to minimize number of duplications yielded similar results (Additional file 4, Figure 2). Thus, very similar estimates of the absolute rate of amino acid substitution were obtained despite the different assumptions made by the tip procedure and the PL method.

The absolute rate of synonymous evolution was estimated using ML estimates of K_S _(Additional file 5A, B). The tip procedure resulted in virtually identical rates for both large and small subunit genes (6.5 × 10^-9 ^synonymous substitutions per synonymous site per year; Additional file 5C). PL rate estimates were 5.5 × 10^-9^± 0.3 × 10^-9 ^and 6.3 × 10^-9 ^± 0.2 × 10^-9 ^(mean ± standard error) synonymous substitutions per synonymous site per year for the large and small subunit, respectively (data not shown). The slightly lower estimates based upon PL are consistent with saturation being a problem, but presumably only for the deepest branches in the tree. All of these values are well within the range of previous estimates for a variety of angiosperm genes (which range from approximately 2 × 10^-9 ^to 10 × 10^-9 ^synonymous substitutions per synonymous site per year and exhibit some variation among lineages [4-7,39,40]). This suggests that there are little to no constraints on the synonymous sites of angiosperm AGPase genes and, when combined with estimate of the absolute rate of sequence evolution, that there was minimal bias in our estimates of ω.

### Does the large subunit show temporary or permanent elevation of ω?

Estimates of the mean rate of evolution for AGPase, whether based upon ω [2] or the absolute rate of amino acid substitution (Figure 2), show a substantially higher rate for the large subunit. The existence of these rate differences despite identical sensitivities to mutations when expressed in bacteria suggests that there are important differences *in planta*. Transient increases in the evolutionary rate might explain the observed rate differences if they are more common for the large subunit. Large-scale analyses provide evidence for transient increase in the rate of non-synonymous evolution. Indeed, paralogs with a recent origin (defined as those with K_S _< 0.1) accumulate more non-synonymous mutations per non-synonymous site (and thus have a higher K_A_) relative to the number of the synonymous mutation per synonymous site than older duplicates [23]. Thus, ω is expected to be elevated for paralogs with K_S _< 0.1 relative to those with K_S _> 0.1. Lynch and Conery [23] interpreted this phenomenon as reflecting a temporary relaxation of constraints, positive selection, or a combination of both phenomena. Thus, it is important to consider the potential impact of the elevation of ω on our analyses of the evolutionary processes that shaped the AGPase gene family.

The large subunit underwent more gene duplications than did the small subunit (Figure 1). Thus, the higher mean estimates of ω for the large subunit might reflect a larger number of periods during which ω is elevated (due to temporary relaxation of constraints and/or positive selection) rather than permanent relaxation of constraints for the large subunit. To distinguish between transient and permanent relaxation of constraint we tested whether the non-synonymous rate was increased after duplication and if the increase is sufficient to explain the observed differences in the mean rate. Branches in both large and small subunit gene trees (Figure 1) were placed into two groups, the first of which (class 1) contained branches that follow a duplication event with K_S _= ~0.1 (these branches are shown in Figure 1B, C and Additional file 3). The second group (class 2) contained all other branches (branches that follow either a speciation event or a duplication and have K_S _> 0.1). We examined two nested models using the likelihood ratio test (LRT) [41-45] to determine whether ω for class 1 branches is higher than ω for class 2 branches for either subunit using CODEML (included in PAML software). The more complex model, which assumes two different ω values (one ω for class 1 and one ω for class 2), was favored over the null hypothesis model, which assumes a single ω for both classes, for both the small subunit (2δ*ln*L = 18.4; *P *< 0.001) and the large subunit (2δ*ln*L = 7.48; *P *= 0.005) (for details see Table 1). The ω estimate for short branches following duplications was 1.3 to 1.5-fold greater than the ω estimate for all the other branches when the large subunit was examined and 2 to 2.8-fold greater for the small subunit (Table 1). These results support periods of increased ω after duplications in the AGPase gene family, due to the temporary relaxation of constraints, positive selection, or both. However, these results also indicate that this phenomenon cannot explain the differential rates of amino acid sequence divergence of the two AGPase subunits, since estimates of ω for the large subunit are 2.6-fold greater than estimates for the small subunit (Table 1). Instead these results suggest that the small subunit is permanently subject to greater purifying selection than is the large subunit.

**Table 1:** Temporary relaxation of purifying selection, positive selection, or both after duplications in the large and small subunit of AGPase from angiosperms.

Branches	Large subunit	p-value	Small subunit	p-value
Class 1 (Figure 1)	0.114	0.005	0.058	< 0.001
Class 2 (Figure 1)	0.086		0.029	
All branches (Figure 1)	0.090		0.033	
				
Class 1 (Additional file 3)	0.125	< 0.001	0.081	< 0.001
Class 2 (Additional file 3)	0.086		0.029	
All branches (Additional file 3)	0.089		0.034	

The branches of the trees shown in Figure 1B and Additional file 3A (large subunit) or Figure 1C and Additional file 3B (small subunit) were classified into two groups. One group includes the short branches (K_S _< 0.1) following a duplication event (post-duplication; class 1) and the other group includes all the other branches (class 2). The overall ω value of each group and the overall ω value of all branches were estimated by CODEML. The LRT was used to compare the model with two different ω values (one for class1 branches and one for class 2 branches) to the null hypothesis model with a single ω value.

### What is the role of positive selection in AGPase evolution?

To examine the potential role of positive selection in AGPase evolution, we used ML to compare two distinct sets of models. The first model set contains a neutral (null) model M1a allowing two categories of sites, one with ω = 0 and one with ω = 1, and model M2a that adds an extra category of sites with ω > 1. The second includes a neutral (null) model M7 assuming that ω is β-distributed among sites and model M8 that adds an extra category of sites with ω > 1 [46]. Neither of the models with positive selection was significantly better than the null model based upon the LRT for either of the subunits when the tests were applied to the complete trees for the small subunit (Figure 1C) or large subunit (Figure 1B) (data not shown). Likewise, neither of the models that include positive selection was significantly better when the tests were applied to the individual groups (Figure 1B, C) within each subunit (groups 1, 2, 3A, and 3B for the large subunit as well as groups 1, and 2 for the small subunit) (data not shown). These results indicate either that positive selection has not played a role in the evolution of the large and small subunit of AGPase in the angiosperms or that these tests have insufficient power.

**Figure 4:**
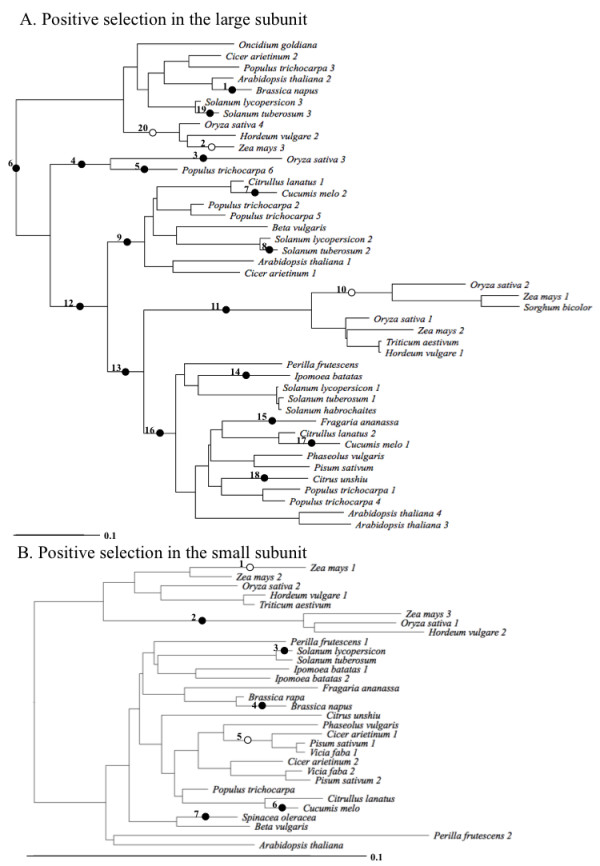
**Positive selection in the large and the small subunit of the angiosperms**. The trees shown in A) and B) have the same topology as the trees shown in Figure 1B and 1C, although the shown here are unrooted. White circles indicate branches where positive selection was detected only by Test 1 but not Test 2 (described in Methods). Black circles indicate branches where positive selection was detected by both Test 1 and Test 2. The branches where positive selection is detected are numbered.

To increase the power of our tests for positive selection, we used branch-site models to examine the potential for positive selection at specific sites in all tree branches separately (Methods). There are branches in the large or small subunit tree on which specific sites may be subject to positive selection (Figure 4), with a total of 0.8 and 0.2 sites/branch potentially affected by positive selection for the large and the small subunit respectively (Additional file 6). However, the limited number of sites potentially affected by positive selection suggests that purifying selection is the major force in the evolution of both AGPase subunits in angiosperms. Thus, positive selection cannot explain the different rates of amino acid evolution for the AGPase subunits.

### Functional divergence of AGPase subunits

Gu [21,22,47] proposed that specific sites in proteins have the potential to undergo two distinct types of divergences after gene duplication (e.g., divergence among the different groups within the large and small subunits), and these types were designated type-I and type-II divergence. Sites that have undergone type-I divergence are conserved in one group but variable in another group while type-II sites are fixed in both groups but differ between groups [21,22].

Tests for types-I and -II divergence based upon the relevant coefficients of divergence (θ), which correspond to the probability that a specific site has undergone type-I or -II divergence in a pairwise comparison, were proposed by Gu [21,22,48]. These tests for types-I and -II establish whether the relevant θ value is significantly greater than zero. We conducted all possible pairwise comparisons among small (groups 1 and 2) and large (groups 1,2, 3a and 3b) subunits with the exception of group 4 of large subunits (that were excluded because the group included only two sequences). All of type-I coefficients (θ_I_) of functional divergence were significantly greater than zero while none of the type-II coefficients (θ_II_) were significantly greater than zero (Table 2). The estimates of θ_I _are also much larger than the estimates of θ_II_, suggesting that type-I divergence is the dominant pattern of sequence evolution for AGPase large and small subunit groups.

**Table 2:** Type I and II functional divergence between large and small subunit groups.

	Large subunit						Small subunit
	
	Group 3b/	Group 3b/	Group 3b/	Group 3a/	Group 3a/	Group 2/	Group 1/
	Group 3a	Group 2	Group 1	Group 2	Group 1	Group 1	Group 2
			Type I divergence				

Theta-I	0.261*	0.383*	0.448*	0.220*	0.331*	0.273*	0.263*
SE	0.069	0.095	0.097	0.060	0.072	0.076	0.084

			TypeII divergence				

Theta-II	0.016	0.072	0.080	0.016	0.058	0.001	0.028
SE	0.061	0.052	0.054	0.059	0.061	0.053	0.033

Coefficients of type I (θ_I_) and II (θ_II_) functional divergence were estimated using DIVERGE. * denotes statistical significance at 5% level of confidence. SE: standard error.

### Sites contributing to functional divergence among AGPase groups

We identified the sites likely to be involved in the changes of functional constraints between groups revealed by the significant values of θ_I _using a posterior probability analysis (Additional file 7). A greater number of large subunit sites appear to have undergone type-I divergence than the number of small subunit sites. Specifically, for an alignment with 453 amino acids we found evidence that 78 large subunit sites and 13 small subunit sites show evidence for type-I divergence (Additional file 8). These sites appear randomly distributed with respect to secondary structure (data not shown), but pairwise comparisons among groups in the large and the small subunit reveal some non-random patterns in the distribution of sites that are conserved in one group but not another. For instance, large subunit group 1 proteins (leaf isoforms) had more conserved sites in the N-terminus while group 3a proteins (sink isoforms) exhibited more conserved in the C-terminus. The large subunit crystal structure has not been elucidated yet. However, the high degree of amino acid sequence identity (~43%) and similarity (~61%) [2] (Additional file 1) to the small subunit along with structure modelling (unpublished data) strongly suggest that the 3D structure of the large subunit is almost identical to the known structure of the small subunit. The N-terminal domain includes the active site that resembles a Rossman fold while the C-terminal domain is a β-helix that extensively interacts with the N-terminal domain based on the structure of the potato tuber small subunit elucidated by Jin et al. [49]. Both domains are important for stability and catalytic/allosteric properties of the enzyme [49-52], but the non-random spatial distribution of type I sites clearly suggests these sites should be targeted in mutational studies focused on the analysis of AGPase structure/function relationships.

In contrast to the significant estimates of θ_I_, estimates of θ_II _(the type-II coefficient) were not significantly greater than zero. However, the power of the test for type-II divergence is unclear. To determine whether there was any evidence for type-II divergence we examined the sequences to determine whether any specific sites show evidence for this type of divergence. The likelihood that these sites reflect *bona fide *instances of type-II divergence would be increased if they correspond to sites that have been identified in mutagenesis studies. The posterior ratio test, using a cut-off 2 (i.e., a posterior probability of 0.5), identified a few potential type-II sites (Additional file 9). Some sites were also identified by analyses of positive selection and one has an important function revealed by mutagenesis (see Discussion), so it is reasonable to postulate that at least some of these sites reflect genuine instances of type-II divergence that have contributed to the specialization among the large and small subunits.

## Discussion

The large and the small subunit of AGPase in plants exhibit considerable sequence identity [1,2] and they reflect a gene duplication that occurred prior to the divergence of land plants and green algae. Both subunits are equally sensitive to activity-altering amino acids, at least when expressed in a bacterial system [2]. However, the small subunit of angiosperms is more conserved than large subunit based upon estimates of the rate of evolution (both estimates of ω [2] and estimates of the absolute rate of amino acid evolution). These results suggest saturation has not had a major impact upon the estimation of ω for angiosperm AGPases, and this may reflect, at least in part, the limited codon bias of these genes (data not shown) since codon bias can have a major impact on the estimation of K_S _[8]. They also suggest that estimation of the absolute rate of amino acid evolution provides a valid method that can be used as an alternative to ω analysis when the use of ω is not appropriate (e.g., ancient divergences, especially for gene families with strong codon bias, and for gene families where there is evidence for strong selection on synonymous sites). Taken as a whole, these results confirm that plant AGPases represent a genuine paradox: the large and small subunits exhibit similar sensitivities to activity-altering changes but differ almost 3-fold in their rates of non-synonymous evolution.

Although a temporary elevation of ω after duplication was observed for both large and small subunit gene families, this transient elevation cannot account for the overall difference in ω value (and the related difference in the overall rate of amino acid substitutions) between the two subunits. Additionally, although both subunits appear to have been subject to positive selection the observed rate differences are too large to be explained by postulating that they reflect greater differences in positive selection. Based upon the falsification of these hypotheses, we conclude that the small subunit has been evolving under stronger purifying selection than the large subunit.

Consistent with the numbers of large and small subunit genes found in the sequenced plant genomes [2,35,38], reconciled tree analyses indicated that large subunit genes underwent more duplications than small subunit genes. Both phylogeny and molecular clock analyses indicate that the initial duplication in the large subunit family of angiosperms occurred *ca*. 400 MY ago, close to the divergence of angiosperms and bryophytes [30] (Figure 3A and Additional file 4A). In contrast, the oldest retained small subunit duplicates date back to 120–140 MY ago, after the divergence of monocots and eudicots (Figure 3B and Additional file 4B). The reason why the large subunit had more ancient and duplications than did the small subunit remains enigmatic. Macroevolutionary models that can be applied to gene families are poorly developed, so it remains possible that the observed difference is coincidental. Alternatively, the large subunit might have a greater ability to undergo subfunctionalization after duplication. The fact that 7 large subunit genes and 4 small subunit genes can be identified in the *Physcomitrella *genome suggests similar patterns in both mosses and angiosperms. However, more rigorous tests to distinguish among these scenarios must await both the acquisition of additional data from additional deep-branching land-plant lineages (e.g., liverworts and hornworts) and the development of better models of gene family macroevolution.

Studies of expression patterns of AGPase genes in several species, including rice, *Arabidopsis*, potato, tomato and barley, have shown that the large subunit is tissue specific while the small subunit is more broadly expressed [33-38]. Based on these studies, the major large subunit groups (Figure 1B) are likely to be expressed in different tissues in most or all plants. The tissue-specificity of large subunit genes suggests the expression patterns of these genes might undergo subfunctionalization after duplication, as predicted by the "DDC model" of gene duplication [53]. The DDC model predicts that duplicated genes are preserved by complementary changes in their expression pattern (e.g., a broadly expressed gene might undergo duplication and have one duplicate expressed in a specific tissue like leaves while the other duplicate is expressed elsewhere). Although the potential for subfunctionalization due to changes in gene expression to preserve duplicated genes is generally accepted [54,55], it also remains possible that distinct AGPase genes have specialized in terms of protein function (e.g., their pH optima might have shifted based upon the specific tissues in which the paralogs are expressed). The relative contributions of subfunctionalization and specialization or neofunctionalization to gene family evolution are open questions [56,57], and it is unclear that there is any reason why the large subunit would be more likely to undergo specialization at either the protein or gene expression level. Such a model, which postulates that subfunctionalization of gene expression was followed by specialization, is similar to a combined model called subneofunctionalization [57]. The subneofunctionalization model postulates that subfunctionalization occurs shortly after duplication while neofunctionalization is a more prolonged process [56,57]. If the combined model were applied to AGPases, the initial preservation of paralogous AGPase genes immediately after duplication might reflect subfunctionalization but this process would be followed by adaptation to the more specialized domains of expression in which each of the paralogs are expressed. Either a neofunctionalization or a subneofunctionalization model would be consistent with the evidence that different large subunit groups have functionally diverged from each other at the protein level (Figure 4, Table 2). However, subneofunctionalization provides a means to directly link the divergence of expression patterns and divergence at the protein level. Corroborating the subneofunctionalization will require correlating gene expression and sequence divergence for a large number of plants.

Differences in their patterns of expression represent the major difference between the large and small subunits that could explain the differences in their rates of evolution, since broadly expressed genes are more conserved than tissue-specific genes [58-60]. However, this raises the question of why broadly expressed genes, like AGPase small subunit genes, exhibit slower rates of evolution. Although it may simply be that mutations in broadly expressed genes have a greater impact on fitness or because these genes have to function in multiple cellular environments [58], a simpler explanation might be that small subunit genes have to function with multiple large subunit genes. Georgelis et al. [2] presented data consistent with this possibility, since they showed that the effects of several amino acid changes in the maize endosperm small subunit on enzyme activity depended on the identity of the large subunit [maize endosperm large subunit (SHRUNKEN-2)(SH2) and maize embryo large subunit (AGPLEMZM) were used]. Both SH2 and AGPLEMZM are members of group 3b (Figure 1B), so these results suggest that even fairly similar large subunit genes can interact differently with small subunits.

In addition to the potential for subfunctionalization due to changes in gene expression, the observation that estimates of θ_I _for large subunit groups were significantly greater than zero suggests that it will be possible to attribute differences among AGPase genes to specific amino acid changes. We found 99 candidate sites of the large subunit likely to have been involved in rate shifts (either type-I or -II divergence; Additional files 8 and 9). At least some of these putative rate shift residues are likely to have contributed to functional changes among the different large subunit groups. The estimate of θ_I _for the small subunit groups 1 (monocot) and 2 (eudicot) is also significantly greater than zero. It was possible to find evidence for 13 type-I candidate sites in the small subunit alignment. Like the large subunit, the estimate of θ_II _for the small subunit was not significantly greater than zero. Nonetheless, there were two potential type-II sites could be identified (Additional file 9).

A total of 21 candidate sites for positive selection could be identified in the large subunit branches following duplications that led to different groups (Branch numbers: 4,6,9,11,12,13,16 shown in Additional file 6), and six of these sites overlapped with the set of sites that appear to have undergone either type-I (sites 341, 364, 445) or type-II (sites 106, 114, 382) divergence. Biochemical and genetic studies confirm that at least one of the sites (sites 106) is important for AGPase activity. This site is a threonine (T) in large subunit groups 3a and 3b but a lysine (K) in groups 1 and 2 and in all small subunits. The potato tuber large subunit, which falls into group 3a, has a T at site 106 and it forms an inactive complex if it is combined with an inactivated potato tuber small subunit [61]. Changing this T in the potato tuber large subunit to a K actually results (the T106K mutant) in a complex with some activity with the same inactivated potato tuber small subunit [61]. These results were interpreted as evidence that the large subunit lost its catalytic ability partly because of the K to T change. Although the K residue at site 106 may be necessary for catalysis if the small subunit is inactive, another model that explains the data would be one in which the wild-type large subunits (which have a T at site 106) require prior catalysis by the small subunit before they perform catalysis by themselves. Such a catalytic mechanism has been proposed for the *Escherichia coli *AGPase [62]. The T residue at site 106 is absolutely conserved in large subunit groups 3 and the branch-site model suggests positive selection for the T immediately following the duplication that generated group 3 (Additional file 6). This suggests that this change was important for enzymatic activity and beneficial for the plants. Indeed, the overall activity of a complex that includes the T106K mutant of the potato tuber large subunit and the wild-type potato tuber small subunit showed significantly reduced activity relative to wild-type potato tuber AGPase [63].

One potential type-II site (site 507) and four sites that represent candidates for positive selection on branches that immediately follow duplications (sites 104, 230, 441, 445) have been shown to be important for the allosteric properties of AGPase [[49,64,65], Hannah personal communication]. However, most of the candidate sites for either rate shifts or positive selection do not have a known function.

The existence of type-I and type-II divergence among AGPase subunit groups along with the detection of positively selected sites after duplications that led to different groups in the large subunit provide evidence for functional divergence especially among the large subunit groups. Our data are consistent with biochemical studies showing that the four possible AGPase complexes in *Arabidopsis*, which have a single functional small subunit gene and four distinct large subunit genes (belonging to different groups in Figure 1B), have different kinetic and allosteric properties [16]. There is further evidence for functional divergence among plant AGPases, since the maize and barley endosperm AGPases are less dependent than potato tuber AGPase on the allosteric activator 3-PGA for activity and the maize endosperm AGPase is more heat labile than potato tuber AGPase [17-20]. Functional divergence among the different subunit groups was also suggested by Georgelis et al. [2], who showed that all groups of large subunit genes have ω values (which range from 0.073 to 0.132) that exceed those for small subunit genes (which range from 0.027 to 0.054). These results are consistent with the rate shifts within the large and the small subunit families that were observed in the present study and they further imply that the various groups of plant AGPases have undergone functional divergence. The present study also identifies specific residues that are likely to have contributed towards that divergence. Site directed mutagenesis of these candidate sites is likely to shed some light on their functions and reveal the proportion of candidate sites that reflect type II error for tests to identify sites subject to rate shifts and positive selection.

A number of angiosperm AGPases have been successfully expressed in *E. coli *and purified, including maize endosperm AGPase [66], potato tuber AGPase [67], and all possible *Arabidopsis *AGPase complexes [16]. The most straightforward candidate sites to test are the type-II sites and those subject to positive selection, where it is possible to change the relevant residue either to the amino acid present in the other group (for type-II sites) or the ancestral amino acid (for positively selected sites). Testing type-I sites may be more challenging, since there is not a clear way to swap the residues present in a member of the focal group of proteins with that in a different group. However, it is reasonable to predict that any of the residues present in the group of proteins for which the site is variable should alter the biochemical activity of a protein in which the site is invariant. Regardless of the specific strategies for generating mutants, mutant large or small subunits could be expressed in *E. coli *along with a wild-type version of the other subunit, the relevant complex purified, and the properties of the enzyme determined to allow the impact of the mutations to be studied. This will allow elucidation of the importance of the sites in enzyme activity. Ultimately, it will be interesting to determine whether these sites are important for the kinetic and allosteric properties of AGPase, for enzyme stability for the pH optimum, or for multiple properties.

## Conclusion

Herein, we validated and extended the observation, initially based upon estimates of ω [2], that the AGPase large subunit accumulated non-synonymous substitutions more rapidly than the small subunit in angiosperms by estimating absolute rates of amino acid change. The earliest duplication in the large subunit family of angiosperms was close to the time that angiosperms and mosses diverged (~400 MY ago;[30]). The large subunit underwent a larger number of duplications than the small subunit, which only began to duplicate after the divergence of monocots and eudicots. We suggest that the large subunit evolved faster due to permanently relaxed constraints since positive selection and sporadic episodes of relaxed constraints cannot account for the different rates of evolution between the large and the small subunit.

Large subunit genes exhibit narrower tissue specificity than small subunit genes in terms of their gene expression patterns [33-38], and they are likely to have experienced subfunctionalization in terms of expression patterns. However, we use analyses of rate shifts and positive selection to demonstrate that different groups of both large and small subunits are likely to have diverged at the protein level. We have identified candidate amino acid sites with the potential to account for the functional divergence and described strategies for site-directed mutagenesis experiments that could shed light into the specific roles of these sites.

## Methods

### Sequence Retrieval and Alignment

Full-length AGPase sequences from plants were retrieved from the NCBI database and the DOE Joint Genome Institute (JGI) web site, and the source and accession numbers of all sequences are presented in Additional file 10. DNA and protein sequence alignments were obtained using the MEGA software [68] with BLOSUM matrix followed by manual inspection. The poorly aligned N-termini (~70–80 amino acids for the large subunit and ~40 amino acids for the small subunit) were excluded from alignment. The large subunit amino acid numbers used correspond to the protein encoded by the maize *Shrunken-2 *(*Sh2*) gene (Accession #: P55241) while the small subunit amino acid numbers used correspond to the protein, encoded by the maize *Brittle-2 *(*Bt2*) gene (Accession #: AAQ14870).

### Phylogenetic analysis

Estimates of AGPase gene trees based upon nucleotide data were obtained using the GARLI (Genetic Algorithm for Rapid Likelihood Inference) software [69]. Estimates of phylogeny estimated for protein sequence alignments were obtained either using neighbor joining in the MEGA software or ML in the RAxML software [70], using the JTT model [71] in both cases (to estimate distances or calculate likelihoods). Bootstrap support [72] was calculated using 100 replicates.

Branch lengths were estimated by ML, with those form amino acid trees based estimated using AAML, using the Dayhoff (PAM) model [73] with Γ-distributed rates. Nonsynonymous substitutions per nonsynonymous site (K_A_) and synonymous substitutions per synonymous site (K_S_) and the ratio of these values (ω = K_A_/K_S_) were estimated using CODEML. AAML and CODEML are programs in the PAML (Phylogenetic Analysis by Maximum Likelihood) package [74].

### Reconciled tree analysis

Reconciled tree analyses map a gene tree onto a species tree [75,76], and most commonly used procedure for doing this is gene tree parsimony, which minimizes the number of duplication events needed to explain a specific gene tree given the species tree [32]. However, some error is typically associated with estimates of phylogeny for individual genes, and accommodating error in gene trees is difficult in reconciled tree analyses [75]. Chen et al. [77] suggested an algorithm that rearranged gene trees to increase congruence with the species tree when nodes in the gene tree were poorly supported to limit the impact of error in the gene tree. We implemented this idea manually, by rearranging nodes in the gene tree with limited support (those with bootstrap support < 70%; see [78]) to increase the congruence with the species tree. This yielded two estimates of the numbers of duplications, one based on the optimal gene trees and a conservative estimate based on well-supported nodes.

### Estimation of the absolute rate of evolution

Estimates of the absolute rate of amino acid evolution and synonymous site evolution for each subunit were obtained using the "tip procedure", which uses the average number of amino acid substitutions per site along unique paths from each tip (extant sequence) to a dated speciation event (allowing us to avoid pseudoreplication of rate estimates). In addition to this method, absolute rates of amino acid substitution were also obtained by estimating the age of each node after smoothing rates using penalized likelihood in r8s ("PL method") [79]. The PL method was used because the tip method is biased towards recent branches, although the PL method also has the potential to be affected by saturation, especially for synonymous sites.

### Detection of branch-specific patterns of rate variation among sites and positive selection

Type-I and type-II functional divergence among large or small subunit groups was examined using the DIVERGE software [48], which implements the tests suggested by Gu [22,47] that can be used to determine whether the coefficients of divergence (θ_I _and θ_II_) are significantly greater than zero. Amino acid sites likely to have undergone types-I or -II divergence were detected as those with a posterior probability > 0.5–0.6.

Sites subject to positive selection were identified using the site, branch and branch-site models implemented in CODEML, using the model comparisons recommended by Yang et al. [80]. The first comparison was between model M1a, which includes two ω values (one for sites subject to purifying selection with ω < 1 and one for neutral sites with ω = 1) and model M2a (which adds positively selected sites [with ω > 1] to model M1a). The second comparison was between model M7, which assumes values of ω at different sites are β-distributed, and model M8 (which adds positively selected sites to model M7). We also searched for positive selection by estimating different values of ω for each branch and using branch-site models. For the first test, we compared a model specified as model = 2 NSsites = 2 to model M1a [81]. For the second test, we compared a model specified as model = 2 NSsites = 2 to a model whose specifications are model = 2 NSsites = 2 fix_omega = 1 and omega = 1. When the likelihood ratio test was significant, the Bayes empirical Bayes method was used to calculate posterior probabilities that sites were subject to positive selection [82].

## Authors' contributions

NG conducted the experiments and carried out the analyses. NG, ELB and LCH conceived and designed the experiments. NG, ELB and LCH wrote the manuscript. All authors read and approved the final manuscript.

## Supplementary Material

Additional file 1 **Alignment of large and small subunits AGPases from angiosperms with protein domains highlighted**. The blue domain indicates the hypervariable N terminus of the large and the small subunit. The pink and green domains indicate the catalytic domain and the β-helix domain respectively. The yellow domain indicates the loop that connects the catalytic to the β-helix domain. Click here for file

Additional file 2 **Species tree**. Times of divergence are indicated in million of years (MY) at nodes [83-92]. All divergence times were examined for consistency with the fossil record [93]. Click here for file

Additional file 3 **Reconciled large and small subunit trees**. A) Angiosperm large subunit reconciled tree. B) Angiosperm small subunit reconciled tree. The topology of the trees shown in A) and B) was determined by ML using aligned cDNA sequences analyzed by GARLI. Nodes with bootstrap values < 70% (Figure 1) were then rearranged to minimize the number of duplications (to increase congruence with the species tree). Branch lengths reflect numbers of amino acid substitutions per site, estimated AAML (with the scale bar showing the number of amino acid substitutions per site). Reconciled tree analyses were conducted using GENETREE and the species tree in Additional file 1. Black boxes indicate duplication events. The trees in A) and B) were rooted with the AGPase large and small subunit from *Chlamydomonas reinhardtii *respectively. Thicker lines indicate branches with K_S _< 0.1 following duplication events (using ML estimates of synonymous branch lengths). Click here for file

Additional file 4 **Phylogenetic trees of the large and the small subunits from angiosperms after rate-smoothing**. The trees in parts A) and B) of this figure are rate-smoothed versions of the gene trees shown in Additional file 3A and 3B that were rearranged to increase congruence with the species tree. Rate-smoothing was done by using the PL method implemented in the r8s software. Black boxes indicate duplication events. Click here for file

Additional file 5 **Average number of synonymous substitutions per site per year**. The trees in A) and B) have the topology of the trees shown in Figure 1B and 1C respectively. The length of the branches represents the number of synonymous substitutions per site as estimated by the free model of CODEML. The bars correspond to the number of synonymous substitutions per site. The numbers of synonymous substitutions per site per year, shown in C), were estimated from the most recent dated speciation events to present sequences of the trees shown in A) and B). The error bars represent 2× standard error. Click here for file

Additional file 6 **Amino acid sites in the large and the small subunit of AGPase from angiosperms under positive selection**. Large subunit site numbers correspond to the amino acid sequence encoded by *Shrunken-2 *(NCBI accession number: P55241). Small subunit site numbers correspond to the amino acid sequence encoded by *Brittle-2 *(NCBI accession number: AAQ14870). Click here for file

Additional file 7 **Distribution of type I sites along the large (A) and the small (B) subunit**. The cut-off value of posterior probability is empirical and it was set to 0.5 for all group comparisons except for group 1-group 3b and group 2-group 3b where the cut-off value was set to 0.6, since theta was greater for these pairs. The Y-axis corresponds to posterior probability. The X-axis corresponds to the number of the amino acid site based on the subunits encoded by *Shrunken-2 *(A) (NCBI accession number: P55241) and *Brittle-2 *(B) (NCBI accession number: AAQ14870). Click here for file

Additional file 8 **Type-I sites in the large and the small subunit of AGPase from angiosperms**. Type-I functional divergence between large and small subunit groups was estimated by DIVERGE. Large subunit site numbers correspond to the amino acid sequence encoded by *Shrunken-2 *(NCBI accession number: P55241). Small subunit site numbers correspond to the amino acid sequence encoded by *Brittle-2 *(NCBI accession number: AAQ14870). Click here for file

Additional file 9 **Type-II sites in the large and the small subunit of AGPase from angiosperms**. Type-II functional divergence between large and small subunit groups was estimated by DIVERGE. Large subunit site numbers correspond to the amino acid sequence encoded by *Shrunken-2 *(NCBI accession number: P55241). Small subunit site numbers correspond to the amino acid sequence encoded by *Brittle-2 *(NCBI accession number: AAQ14870). Click here for file

Additional file 10 AGPase subunit accession numbers Click here for file
