# Supplementary material for: Duplications and functional divergence of ADP-glucose pyrophosphorylase genes in plants
Source: BMC Evol Biol. 2008 Aug 12;8:232. doi: 10.1186/1471-2148-8-232 (PMC2529307; doi:10.1186/1471-2148-8-232)
Supplement: Additional file 1 — Alignment of large and small subunits AGPases from angiosperms with protein domains highlighted. The blue domain indicates the hypervariable N terminus of the large and the small subunit. The pink and green domains indicate the catalytic domain and the β-helix domain respectively. The yellow domain indicates the loop that connects the catalytic to the β-helix domain. [file 1471-2148-8-232-S1.pdf]

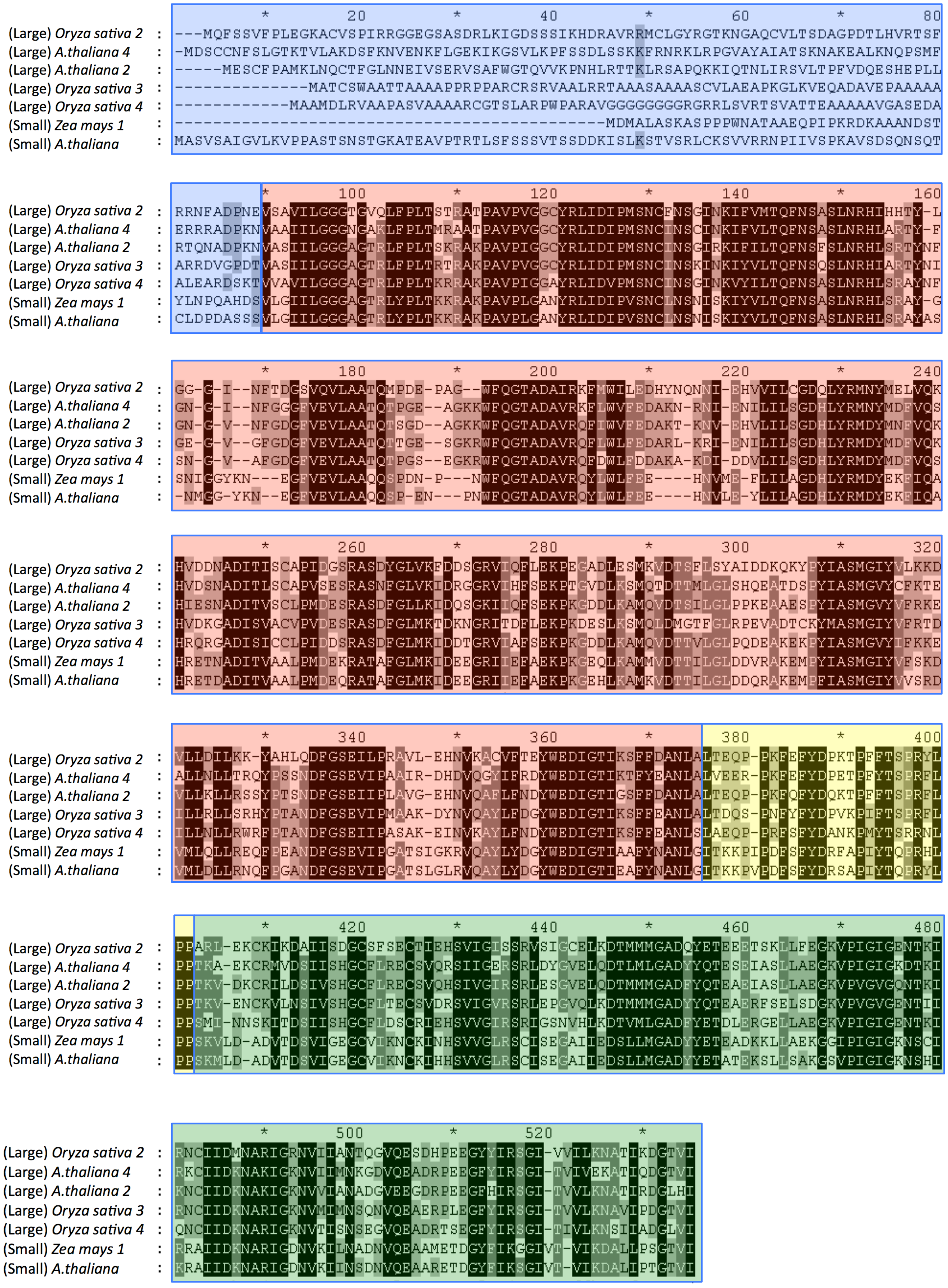
* 20 * 40 * 60 * 80
(Large) Oryza sativa 2 : ---MQFSSVFPLEGKACVSPIRRGGEGSASDRLKIGDSSSIKHDRAVRRMCLGYRGTKNGAQCVLTSDAGPDTLHVRTSF
(Large) A.thaliana 4 : -MDSCCNFSLGTKTVLAKDSFKNVENKFLGEKIKGSVLKPFSSDLSSKKFRNRKLRPGVAYAIATSKNAKEALKNQPSMF
(Large) A.thaliana 2 : -----MESCFPAMKLNQCTFGLNNEIVSERVSAFWGTQVVKPNHLRTTKLRSAPQKKIQTNLIRSVLTPFVDQESHEPLL
(Large) Oryza sativa 3 : --------------MATCSWAATTAAAAPPRPPARCRSRVAALRRTAAASAAAASCVLAEAPKGLKVEQADAVEPAAAAA
(Large) Oryza sativa 4 : ------------MAAMDLRVAAPASVAAAARCGTSLARPWPARAVGGGGGGGGRGRRLSVRTSVATTEAAAAAVGASEDA
(Small) Zea mays 1 : ---MDMALASKASPPPWNATAAEQPIPKRDKAAANDST
(Small) A.thaliana : MASVSAIGVLKVPPASTSNSTGKATEAVPTRTLSFSSSVTSSDDKISLKSTVSRLCKSVVRRNPIIVSPKAVSDSQNSQT
* 100 * 120 * 140 * 160
(Large) Oryza sativa 2 : RRNFADPNEVSAVILGGGTGVQLFPLTSTRATPAVPVGGCYRLIDIPMSNCFNSGINKIFVMTQFNSASLNRHIHHTY-L
(Large) A.thaliana 4 : ERRRADPKNVAAIILGGGNGAKLFPLTMRAATPAVPVGGCYRLIDIPMSNCINSCINKIFVLTQFNSASLNRHLARTY-F
(Large) A.thaliana 2 : RTQNADPKNVASIILGGGAGTRLFPLTSKRAKPAVPVGGCYRLIDIPMSNCINSGIRKIFILTQFNSFSLNRHLSRTYNF
(Large) Oryza sativa 3 : ARRDVGPDTVASIILGGGAGTRLFPLTKRRAKPAVPVGGCYRLIDIPMSNCINSKINKIYVLTQFNSQSLNRHIARTYNI
(Large) Oryza sativa 4 : ALEARDSKTVVAVILGGGAGTRLFPLTKRRAKPAVPIGGAYRLIDVPMSNCINSGINKVYILTQFNSASLNRHLSRAYNF
(Small) Zea mays 1 : YLNPQAHDSVLGIILGGGAGTRLYPLTKKRAKPAVPLGANYRLIDIPVSNCLNSNISKIYVLTQFNSASLNRHLSRAY-G
(Small) A.thaliana : CLDPDASSSVLGIILGGGAGTRLYPLTKKRAKPAVPLGANYRLIDIPVSNCLNSNISKIYVLTQFNSASLNRHLSRAYAS
* 180 * 200 * 220 * 240
(Large) Oryza sativa 2 : GG-G-I--NFTDGSVQVLAATQMPDE-PAG--WFQGTADAIRKFMWILEDHYNQNNI-EHVVILCGDQLYRMNYMELVQK
(Large) A.thaliana 4 : GN-G-I--NFGGGFVEVLAATQTPGE--AGKKWFQGTADAVRKFLWVFEDAKN-RNI-ENILILSGDHLYRMNYMDFVQS
(Large) A.thaliana 2 : GN-G-V--NFGDGFVEVLAATQTSGD--AGKKWFQGTADAVRQFIWVFEDAKT-KNV-EHVLILSGDHLYRMDYMNFVQK
(Large) Oryza sativa 3 : GE-G-V--GFGDGFVEVLAATQTTGE--SGKRWFQGTADAVRQFLWLFEDARL-KRI-ENILILSGDHLYRMDYMDFVQK
(Large) Oryza sativa 4 : SN-G-V--AFGDGFVEVLAATQTPGS--EGKRWFQGTADAVRQFDWLFDDAKA-KDI-DDVLILSGDHLYRMDYMDFVQS
(Small) Zea mays 1 : SNIGGYKN---EGFVEVLAAQQSPDN-P---NWFQGTADAVRQYLWLFEE----HNVME-FLILAGDHLYRMDYEKFIQA
(Small) A.thaliana : -NMGG-YKN--EGFVEVLAAQQSP-EN---PNWFQGTADAVRQYLWLFEE----HNVLE-YLILAGDHLYRMDYEKFIQA
* 260 * 280 * 300 * 320
(Large) Oryza sativa 2 : HVDDNADITISCAPIDGSRASDYGLVKFDDSGRVIQFLEKPEGADLESMKVDTSFLSYAIDDKQKYPYIASMGIYVLKKD
(Large) A.thaliana 4 : HVDSNADITLSCAPVSESRASNFGLVKIDRGGRVIHFSEKPTGVDLKSMQTDTTMLGLSHQEATDSPYIASMGVYCFKTE
(Large) A.thaliana 2 : HIESNADITVSCLPMDESRASDFGLLKIDQSGKIIQFSEKPKGDDLKAMQVDTSILGLPPKEAAESPYIASMGVYVFRKE
(Large) Oryza sativa 3 : HVDKGADISVACVPVDESRASDFGLMKTDKNGRITDFLEKPKDESLKSMQLDMGTFGLRPEVADTCKYMASMGIYVFRTD
(Large) Oryza sativa 4 : HRQRGADISICCLPIDDSRASDFGLMKIDDTGRVIAFSEKPKGDDLKAMQVDTTVLGLPQDEAKEKPYIASMGVYIFKKE
(Small) Zea mays 1 : HRETNADITVAALPMDEKRATAFGLMKIDEEGRIIEFAEKPKGEQLKAMMVDTTILGLDDVRAKEMPYIASMGIYVFSKD
(Small) A.thaliana : HRETDADITVAALPMDEQRATAFGLMKIDEEGRIIEFAEKPKGEHLKAMKVDTTILGLDDQRAKEMPFIASMGIYVVSRD
* 340 * 360 * 380 * 400
(Large) Oryza sativa 2 : VLLDILKK-YAHLQDFGSEILPRAVL-EHNVKACVFTEYWEDIGTIKSFFDANLALTEQP-PKFEFYDPKTPFFTSPRYL
(Large) A.thaliana 4 : ALLNLLTRQYPSSNDFGSEVIPAAIR-DHDVQGYIFRDYWEDIGTIKTFYEANLALVEER-PKFEFYDPETPFYTSPRFL
(Large) A.thaliana 2 : VLLKLLRSSYPTSNDFGSEIIPLAVG-EHNVQAFLFNDYWEDIGTIGSFFDANLALTEQP-PKFQFYDQKTPFFTSPRFL
(Large) Oryza sativa 3 : ILLRLLSRHYPTANDFGSEVIPMAAK-DYNVQAYLFDGYWEDIGTIKSFFEANLALTDQS-PNFYFYDPVKPIFTSPRFL
(Large) Oryza sativa 4 : ILLNLLRWRFPTANDFGSEIIPASAK-EINVKAYLFNDYWEDIGTIKSFFEANLSLAEQP-PRFSFYDANKPMYTSRRNL
(Small) Zea mays 1 : VMLQLLREQFPEANDFGSEVIPGATSIGKRVQAYLYDGYWEDIGTIAAFYNANLGITKKPIPDFSFYDRFAPIYTQPRHL
(Small) A.thaliana : VMLDLLRNQFPGANDFGSEVIPGATSLGLRVQAYLYDGYWEDIGTIEAFYNANLGITKKPVPDFSFYDRSAPIYTQPRYL
* 420 * 440 * 460 * 480
(Large) Oryza sativa 2 : PPARI-EKCKIKDAIISDGCSFSECTIEHSVIGISSRVSIGCELKDTMMMGADQYETEEETSKLLFEGKVPIGIGENTKI
(Large) A.thaliana 4 : PPTKA-EKCRMVDSIISHGCFLRECSVQRSIIGERSRLDYGVELQDTLMLGADYYQTESEIASLLAEGKVPIGIGKDTKI
(Large) A.thaliana 2 : PPTKV-DKCRILDSIVSHGCFLRECSVQHSIVGIRSRLESGVELQDTMMMGADFYQTEAEIASLLAEGKVPVGVGQNTKI
(Large) Oryza sativa 3 : PPTKV-ENCKVLNSIVSHGCFLTECSVDRSVIGVRSRLEPGVQLKDTMMMGADYYQTEAERFSELSDGKVPVGVGENTII
(Large) Oryza sativa 4 : PPSMI-NNSKITDSIISHGCFLDSCRIEHSVVGIRSRIGSNVHLKDTVMLGADFYETDLERGELLAEGKVPIGIGENTKI
(Small) Zea mays 1 : PPSKVLD-ADVTDSVIGEGCVIKNCKINHSVVGLRSCISEGAIIEDSLLMGADYYETEADKKLLAEKGGIPIGIGKNSCI
(Small) A.thaliana : PPSKMLD-ADVTDSVIGEGCVIKNCKIHHSVVGLRSCISEGAIIEDSLLMGADYYETATEKSLLSAKGSVPIGIGKNSHI
* 500 * 520 *
(Large) Oryza sativa 2 : RNCIIDMNARIGRNVIIANTQGVQESDHPEEGYYIRSGI-VVILKNATIKDGTVI
(Large) A.thaliana 4 : RKCIIDKNAKIGKNVIIMNKGDVQEADRPEEGFYIRSGI-TVIVEKATIQDGTVI
(Large) A.thaliana 2 : KNCIIDKNAKIGKNVVIANADGVEEGDRPEEGFHIRSGI-TVVLKNATIRDGLHI
(Large) Oryza sativa 3 : RNCIIDKNARIGKNVMIMNSQNVQEAERPLEGFYIRSGI-TVVLKNAVIPDGTVI
(Large) Oryza sativa 4 : QNCIIDKNARIGKNVTISNSEGVQEADRTSEGFYIRSGI-TIVLKNSIIADGLVI
(Small) Zea mays 1 : RRAIIDKNARIGDNVKILNADNVQEAAMETDGYFIKGGIVT-VIKDALLPSGTVI
(Small) A.thaliana : KRAIIDKNARIGDNVKIINSDNVQEAARETDGYFIKSGIVT-VIKDALIPTGTVI
