# Supplementary figures and images for: Duplications and functional divergence of ADP-glucose pyrophosphorylase genes in plants
Source: BMC Evol Biol. 2008 Aug 12;8:232. doi: 10.1186/1471-2148-8-232 (PMC2529307; doi:10.1186/1471-2148-8-232)

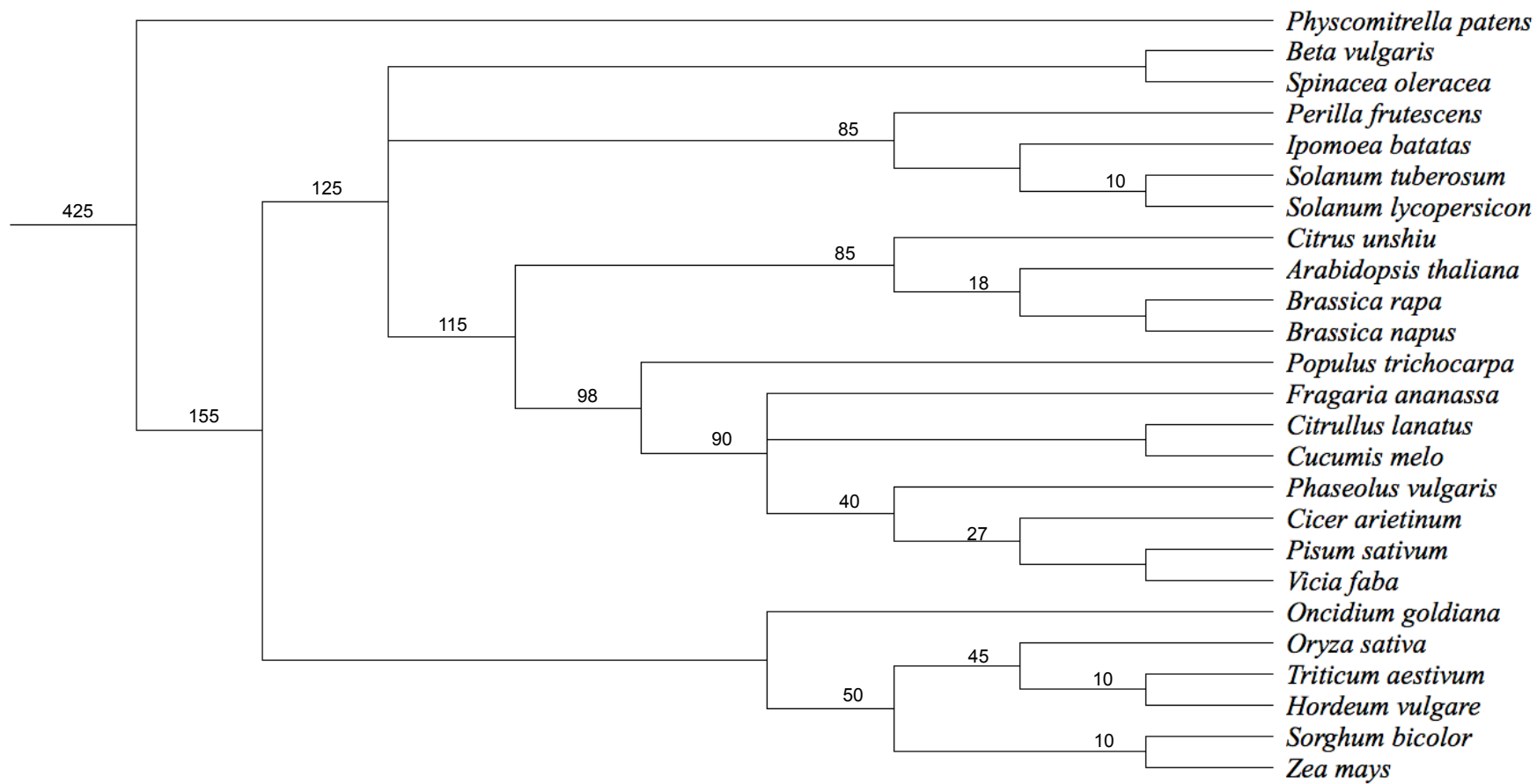

Supplement: Additional file 2 — Species tree. Times of divergence are indicated in million of years (MY) at nodes [83-92]. All divergence times were examined for consistency with the fossil record [93]. [file 1471-2148-8-232-S2.pdf]

## A Large subunit reconciled tree

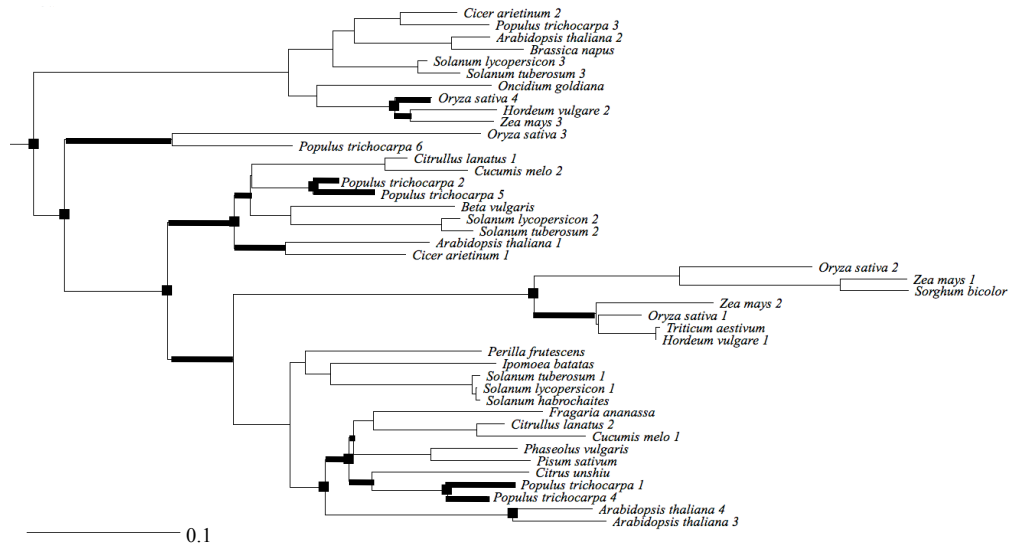

## B Small subunit reconciled tree

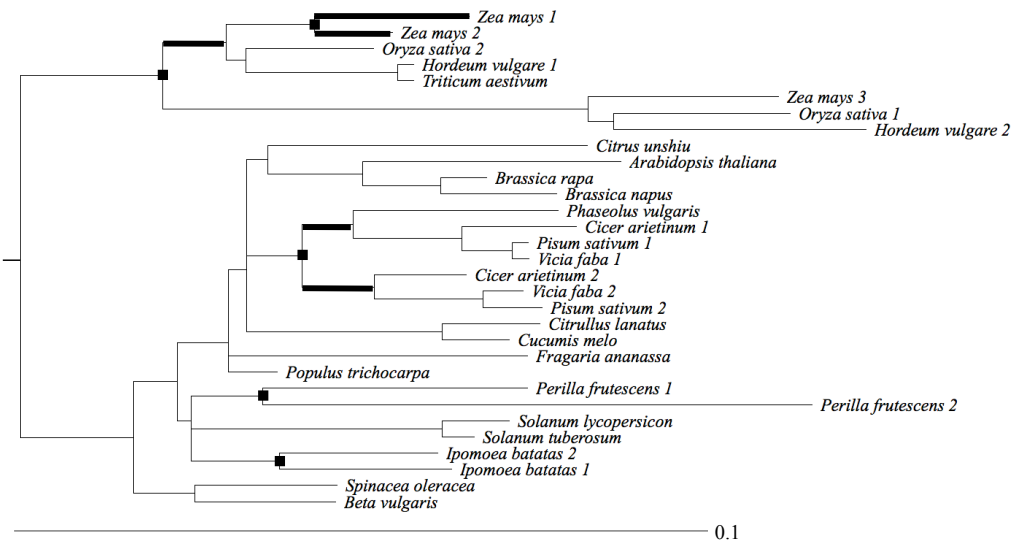

Supplement: Additional file 3 — Reconciled large and small subunit trees. A) Angiosperm large subunit reconciled tree. B) Angiosperm small subunit reconciled tree. The topology of the trees shown in A) and B) was determined by ML using aligned cDNA sequences analyzed by GARLI. Nodes with bootstrap values < 70% (Figure 1) were then rearranged to minimize the number of duplications (to increase congruence with the species tree). Branch lengths reflect numbers of amino acid substitutions per site, estimated AAML (with the scale bar showing the number of amino acid substitutions per site). Reconciled tree analyses were conducted using GENETREE and the species tree in Additional file 1. Black boxes indicate duplication events. The trees in A) and B) were rooted with the AGPase large and small subunit from Chlamydomonas reinhardtii respectively. Thicker lines indicate branches with KS < 0.1 following duplication events (using ML estimates of synonymous branch lengths). [file 1471-2148-8-232-S3.pdf]

A.

Group 3a/ Group 2

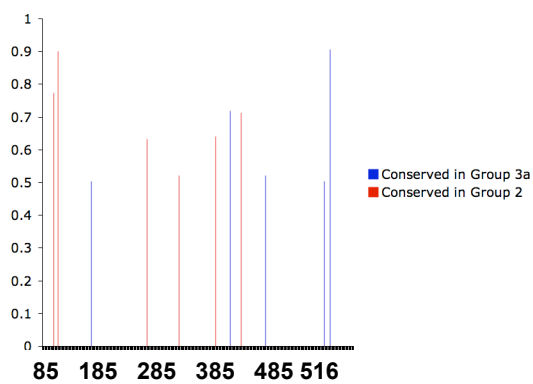

Group 3a/ Group 1

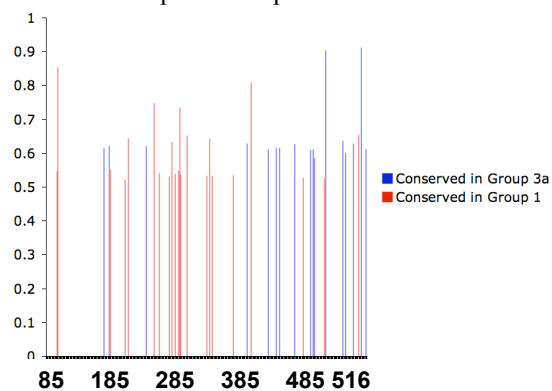

Group 2/ Group 1

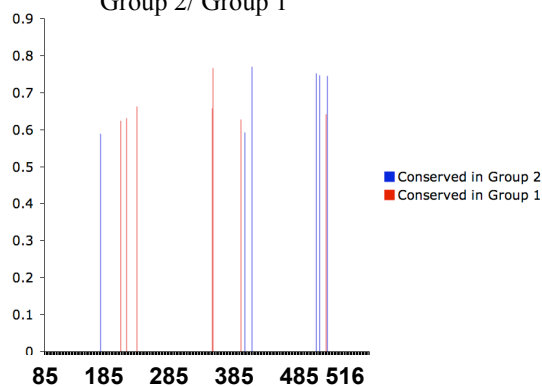

Group 3b/ Group 3a

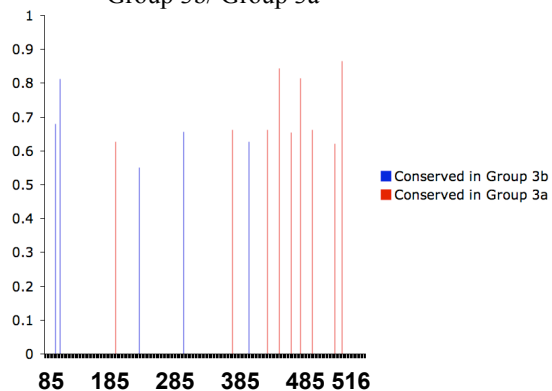

Group 3b/ Group 2

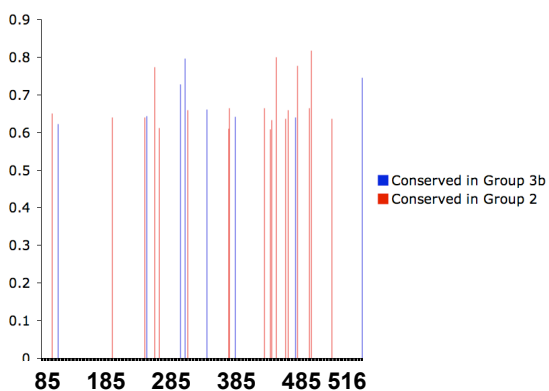

Group 3b/ Group 1

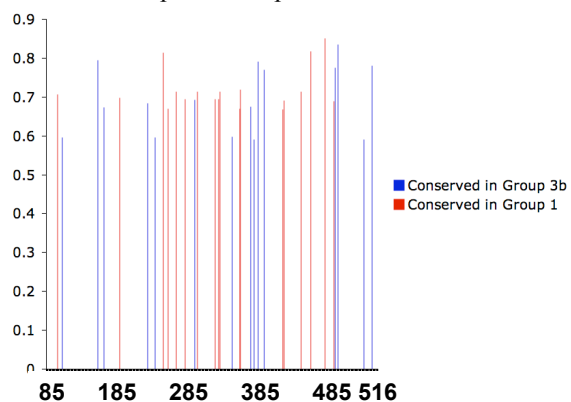

B.

Group 1/ Group 2

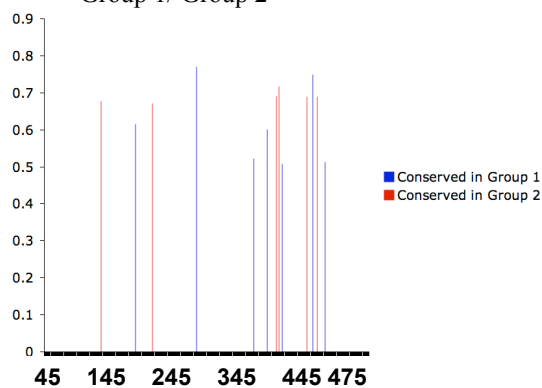

Supplement: Additional file 7 — Distribution of type I sites along the large (A) and the small (B) subunit. The cut-off value of posterior probability is empirical and it was set to 0.5 for all group comparisons except for group 1-group 3b and group 2-group 3b where the cut-off value was set to 0.6, since theta was greater for these pairs. The Y-axis corresponds to posterior probability. The X-axis corresponds to the number of the amino acid site based on the subunits encoded by Shrunken-2 (A) (NCBI accession number: P55241) and Brittle-2 (B) (NCBI accession number: AAQ14870). [file 1471-2148-8-232-S7.pdf]
