# Supplementary material for: Duplications and functional divergence of ADP-glucose pyrophosphorylase genes in plants
Source: BMC Evol Biol. 2008 Aug 12;8:232. doi: 10.1186/1471-2148-8-232 (PMC2529307; doi:10.1186/1471-2148-8-232)

A Large subunit amino acid tree after rate-smoothing

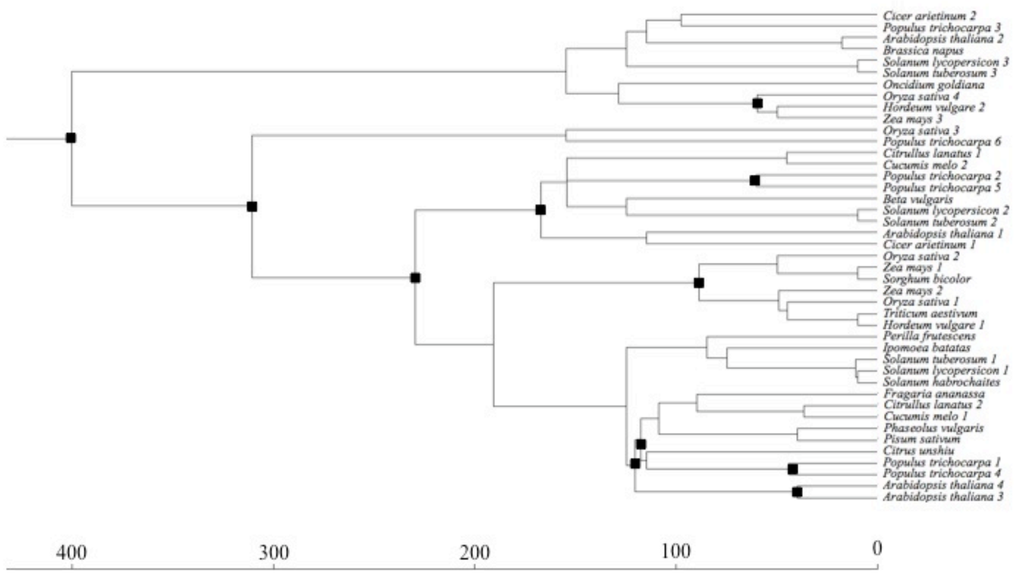

B Small subunit amino acid tree after rate-smoothing

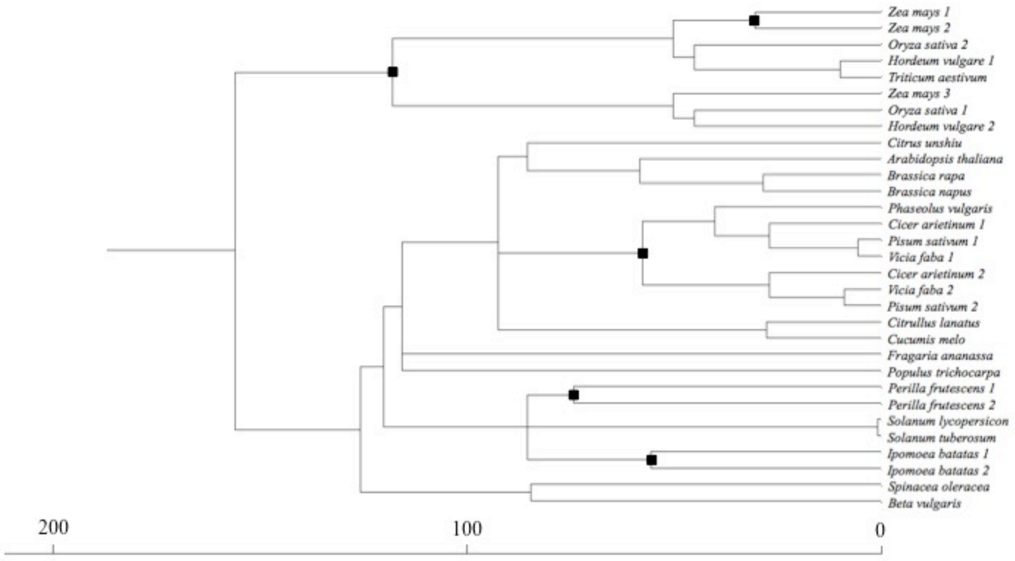

Supplement: Additional file 4 — Phylogenetic trees of the large and the small subunits from angiosperms after rate-smoothing. The trees in parts A) and B) of this figure are rate-smoothed versions of the gene trees shown in Additional file 3A and 3B that were rearranged to increase congruence with the species tree. Rate-smoothing was done by using the PL method implemented in the r8s software. Black boxes indicate duplication events. [file 1471-2148-8-232-S4.pdf]
