# Supplementary material for: Duplications and functional divergence of ADP-glucose pyrophosphorylase genes in plants
Source: BMC Evol Biol. 2008 Aug 12;8:232. doi: 10.1186/1471-2148-8-232 (PMC2529307; doi:10.1186/1471-2148-8-232)

A Large subunit synonymous tree

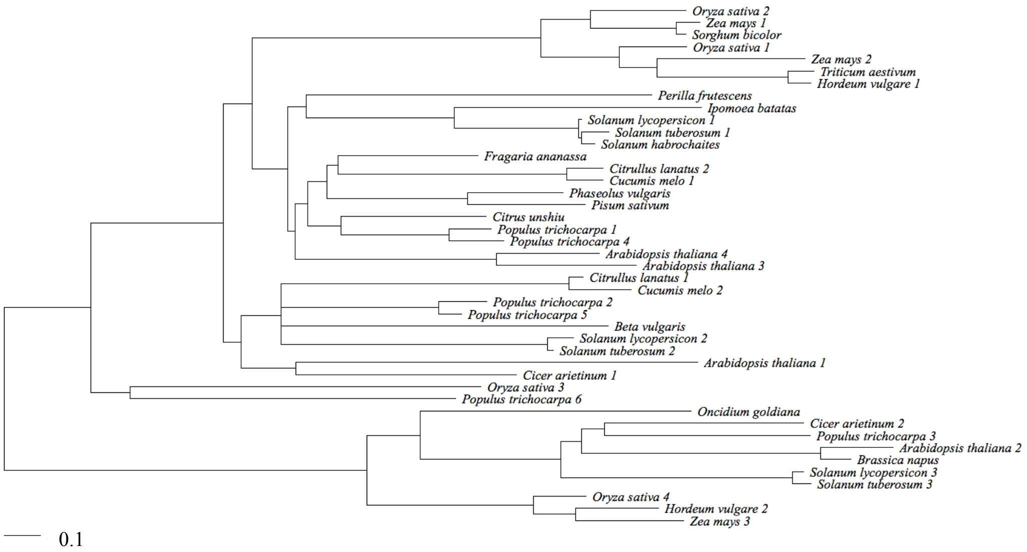

B Small subunit synonymous tree

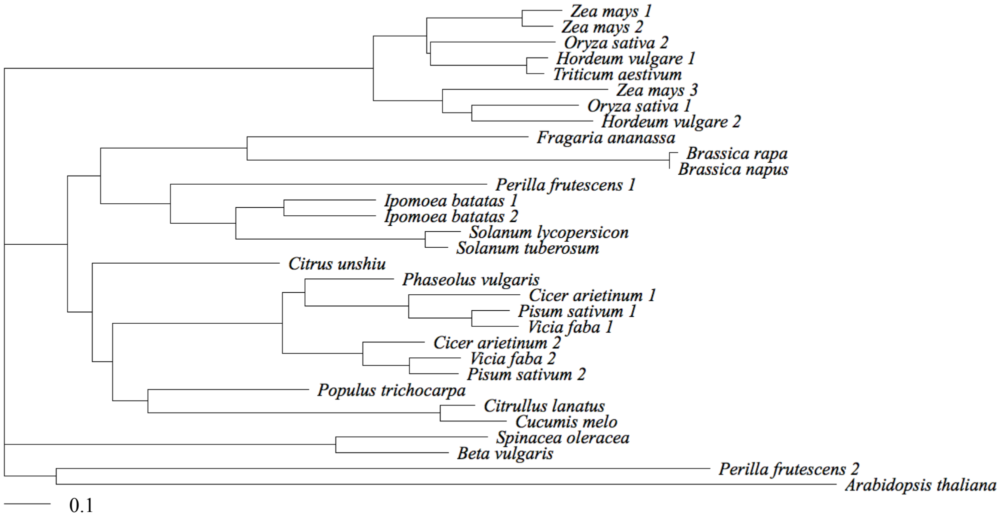

C Number of synonymous substitutions per synonymous site per year (X 10^{-9})

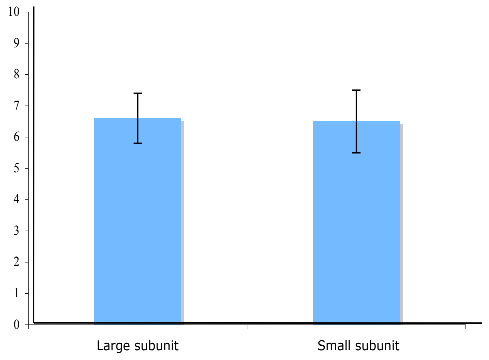

Supplement: Additional file 5 — Average number of synonymous substitutions per site per year. The trees in A) and B) have the topology of the trees shown in Figure 1B and 1C respectively. The length of the branches represents the number of synonymous substitutions per site as estimated by the free model of CODEML. The bars correspond to the number of synonymous substitutions per site. The numbers of synonymous substitutions per site per year, shown in C), were estimated from the most recent dated speciation events to present sequences of the trees shown in A) and B). The error bars represent 2× standard error. [file 1471-2148-8-232-S5.pdf]
