# Supplementary material for: Duplications and functional divergence of ADP-glucose pyrophosphorylase genes in plants
Source: BMC Evol Biol. 2008 Aug 12;8:232. doi: 10.1186/1471-2148-8-232 (PMC2529307; doi:10.1186/1471-2148-8-232)
Supplement: Additional file 6 — Amino acid sites in the large and the small subunit of AGPase from angiosperms under positive selection. Large subunit site numbers correspond to the amino acid sequence encoded by Shrunken-2 (NCBI accession number: P55241). Small subunit site numbers correspond to the amino acid sequence encoded by Brittle-2 (NCBI accession number: AAQ14870). [file 1471-2148-8-232-S6.pdf]

| Branch# | Large subunit | Small subunit |
|---|---|---|
| 1 | 227, 228, 229, 231, 390, 460, 461, 462 | 420 |
| 2 | 142, 486 | 242 |
| 3 | 284, 292, 444, 446 | 413 |
| 4 | 142, 509 | 67, 68, 202, 248, 330 |
| 5 | 153 | 369 |
| 6 | 114, 227, 261, 382 | 78 |
| 7 | 134, 135, 136, 138, 383, 412 | 412 |
| 8 | 169, 178, 258, 441 | |
| 9 | 131, 142 | |
| 10 | 361 | |
| 11 | 155, 160, 187, 198, 424, 441, 445 | |
| 12 | 230, 368 | |
| 13 | 106 | |
| 14 | 152, 436, 465 | |
| 15 | 94, 411, 449, 469 | |
| 16 | 104, 341, 364 | |
| 17 | 160, 273, 363 | |
| 18 | 112, 366, 368, 394 | |
| 19 | 161, 355, 444, 498 | |
| 20 | 175 | |
