# Supplementary material for: Duplications and functional divergence of ADP-glucose pyrophosphorylase genes in plants
Source: BMC Evol Biol. 2008 Aug 12;8:232. doi: 10.1186/1471-2148-8-232 (PMC2529307; doi:10.1186/1471-2148-8-232)
Supplement: Additional file 8 — Type-I sites in the large and the small subunit of AGPase from angiosperms. Type-I functional divergence between large and small subunit groups was estimated by DIVERGE. Large subunit site numbers correspond to the amino acid sequence encoded by Shrunken-2 (NCBI accession number: P55241). Small subunit site numbers correspond to the amino acid sequence encoded by Brittle-2 (NCBI accession number: AAQ14870). [file 1471-2148-8-232-S8.pdf]

Large subunit						Small subunit
Group 3a/ Group 2	Group 3a/ Group 1	Group 2/ Group 1	Group 3b/ Group 3a	Group 3b/ Group 2	Group 3b/ Group 1	Group 1/ Group 2
96	95	163	96	95	95	123
102	96	193	102	103	102	165
151	166	206	187	187	149	187
241	172	218	225	235	163	250
281	173	312	280	238	187	333
330	201	313	342	247	229	352
350	207	350	364	253	238	364
364	232	355	387	280	247	367
398	241	366	403	285	253	373
475	247	445	417	288	263	408
482	259	451	429	312	273	417
	263	458	443	341	285	423
	267	461	473	342	288	435
	271		482	350	309	
	272			387	313	
	273			395	317	
	281			397	333	
	306			403	341	
	309			414	342	
	312			417	355	
	341			427	359	
	359			429	366	
	364			443	373	
	387			448	395	
	398			473	397	
	402			515	417	
	421				429	
	430				448	
	440				458	
	443				461	
	445				464	
	458				497	
	461				509	
	482					
	485					
	497					
	505					
	509					
	514					
