# Supplementary material for: Duplications and functional divergence of ADP-glucose pyrophosphorylase genes in plants
Source: BMC Evol Biol. 2008 Aug 12;8:232. doi: 10.1186/1471-2148-8-232 (PMC2529307; doi:10.1186/1471-2148-8-232)
Supplement: Additional file 9 — Type-II sites in the large and the small subunit of AGPase from angiosperms. Type-II functional divergence between large and small subunit groups was estimated by DIVERGE. Large subunit site numbers correspond to the amino acid sequence encoded by Shrunken-2 (NCBI accession number: P55241). Small subunit site numbers correspond to the amino acid sequence encoded by Brittle-2 (NCBI accession number: AAQ14870). [file 1471-2148-8-232-S9.pdf]

Large subunit						Small subunit
Group 3b/ Group 3a	Group 3b/ Group 2	Group 3b/ Group 1	Group 3a/ Group 2	Group 3a/ Group 1	Group 2/ Group 1	Group 1/ Group 2
172	96	96	106	106	374	232
506	106	106	506	114	438	347
507	163	151	507	151		
	172	213		336		
	213	372		372		
	438	374		374		
	444	380		380		
	502	396		382		
		502		396		
				416		
				425		
				506		
				507		
				508		
