# Supplementary material for: Duplications and functional divergence of ADP-glucose pyrophosphorylase genes in plants
Source: BMC Evol Biol. 2008 Aug 12;8:232. doi: 10.1186/1471-2148-8-232 (PMC2529307; doi:10.1186/1471-2148-8-232)
Supplement: Additional file 10 — AGPase subunit accession numbers [file 1471-2148-8-232-S10.pdf]

Supplemental Table 1
AGPase subunits

Accession	Name	Tissue(s) of expression	Reference(s)
		Cyanobacterial AGPase	
S24991	*Anabaena* sp.PCC 7120	-	43
		Small subunits	
P30523	*Triticum aestivum*	seed endosperm	1
AAQ14870	*Zea mays 1*	seed endosperm	2
AAK69628	*Zea mays 2*	leaf	3
AAK39640	*Zea mays 3*	seed embryo	3
CAA88449	*Hordeum vulgare 1*	seed endosperm	4,5
AAO16183	*Hordeum vulgare 2*	seed endosperm	4
XP_481806	*Oryza sativa 2*	seed endosperm	6,7
AAK27313	*Oryza sativa 1*	seed endosperm, leaf	6,7,8
AAK27721	*Cicer arietinum 1*	seed, weak in leaf	9
AAK27720	*Cicer arietinum 2*	seed, weak in leaf	9
CAB89863	*Brassica napus*	leaf, seed	10
AAK27684	*Brassica rapa*	leaf	11
CAA65540	*Pisum sativum 1*	seed, weak in leaf,stem,root	12
CAA65539	*Pisum sativum 2*	seed, leaf	12
CAA54259	*Vicia faba 1*	seed	13
CAA54260	*Vicia faba 2*	leaf,seed	13
BAC66693	*Phaseolus vulgaris*	seed	14
P23509	*Solanum tuberosum*	tuber,leaf	15,16
CAA58473	*Ipomoea batatas 1*	tuber,leaf	17
CAB01912	*Ipomoea batatas 2*	tuber,stem,leaf	18
AAB00482	*Lycopersicon esculentum*	leaf,fruit	19
CAA58475	*Spinacea oleracia*	leaf	No publication
AAF66434	*Perilla frutescens 1*	stem, root, seed, leaf	20
AAF66435	*Perilla frutescens 2*	stem, root, seed, leaf	20
AAB09585	*Arabidopsis thaliana*	leaf,stem, inflorescence	21,23,27
AAB91466	*Citrullus lanatus*	fruit	23
AAS00541	*Fragaria ananassa*	Unknown	No publication
AAD56041	*Citrus unshiu*	fruit, leaf	24
CAA55515	*Beta vulgaris*	leaves,weak in hypocotyl,tap root, root	25
AAB91462	*Cucumis melo*	leaf,early fruit, weak in root and stem	26
573143 (Protein ID in JGI)	*Populus trichocarpa*	Unknown	No publication
AAF75832	*Chlamydomonas reinhardtii*	-	42
AAS88879	*Ostreococcus tauri*	-	29
32753 (Protein ID in JGI)	*Ostreococcus lucimarinus*	-	No publication
54996 (Protein ID in JGI)	*Physcomitrella patens 1*	Unknown	No publication
108225 (Protein ID in JGI)	*Physcomitrella patens 2*	Unknown	No publication
111529 (Protein ID in JGI)	*Physcomitrella patens 3*	Unknown	No publication
184719 (Protein ID in JGI)	*Physcomitrella patens 4*	Unknown	No publication

		Large subunits	
CAA79980	*Triticum aestivum*	seed endosperm	1,28
P55241	*Zea mays 1*	seed endosperm	30
CAA86227	*Zea mays 2*	embryo	31
Shaw and Hannah unpublished	*Zea mays 3*	leaf	No publication
AAB94012	*Sorghum bicolor*	seed endosperm	19
CAA47626	*Hordeum vulgare 1*	seed endosperm	32,33
AAC49729	*Hordeum vulgare 2*	leaf, weak in endosperm	32
BAA23490	*Oryza sativa 1*	seed endosperm	6,7
AAB38781	*Oryza sativa 2*	seed endosperm	6,7
AAT78793	*Oryza sativa 4*	leaf	6,7
NP_911710	*Oryza sativa 3*	stem,leaf,seed	6,7
AAK27718	*Cicer arietinum 2*	leaf	9
AAK27719	*Cicer arietinum 1*	seeds,weak in leaf,stem and root	9
AAK27685	*Brassica rapa*	leaf	11
CAA65541	*Pisum sativum*	sink tissues:seed,pod,seed coat	12
BAC66692	*Phaseolus vulgaris*	seed	14
CAA53741	*Solanum tuberosum 3*	tuber	34
CAA52917	*Solanum tuberosum 2*	leaf, tuber	34
CAA43490	*Solanum tuberosum 1*	tuber,weak in leaf	35
AAC21562	*Ipomoea batatas*	tuber, stem	36
AAC49941	*Lycopersicon esculentum 2*	stems,roots,early fruit	37
AAC49943	*Lycopersicon esculentum 3*	leaf	37
AAC49942	*Lycopersicon esculentum 1*	fruit, root	37
AAD56405	*Lycopersicon hirsutum*	fruit	38
AAF66436	*Perilla frutescens*	stem,cotyledon	20
AAM14190	*Arabidopsis thaliana 1*	low levels of expression	22
AAM20291	*Arabidopsis thaliana 3*	sink tissues	22
CAA77173	*Arabidopsis thaliana 4*	sink tissues	39
BAA76362	*Arabidopsis thaliana 2*	leaf	40
AAB91468	*Citrullus lanatus 1*	fruit	23
AAB91467	*Citrullus lanatus 2*	fruit	23
AAS00542	*Fragaria ananassa*	Unknown	No publication
AAD56042	*Citrus unshiu*	fruit, leaf	24
CAA55516	*Beta vulgaris*	leaf,weak in hypoc,root	25
AAB91463	*Cucumis melo 1*	stem,root,early fruit, weak in leaf	26
AAB91464	*Cucumis melo 2*	stem,root,early fruit, weak in leaf	26
AAM95945	*Oncidium goldiana*	leaf, flower	41
720391 (Protein ID in JGI)	*Populus trichocarpa 1*	Unknown	No publication
346791 (Protein ID in JGI)	*Populus trichocarpa 2*	Unknown	No publication
721986 (Protein ID in JGI)	*Populus trichocarpa 3*	Unknown	No publication
546089 (Protein ID in JGI)	*Populus trichocarpa 4*	Unknown	No publication
685406 (Protein	*Populus trichocarpa 5*	Unknown	No publication

ID in JGI)			
57133 (Protein ID in JGI)	*Populus trichocarpa 6*	Unknown	No publication
187891 (Protein ID in JGI)	*Chlamydomonas reinhardtii*	Unknown	42
AAS88891	*Ostreococcus tauri*	Unknown	29
42209 (Protein ID in JGI)	*Ostreococcus lucimarinus*	Unknown	No publication
64229 (Protein ID in JGI)	*Physcomitrella patens 1*	Unknown	No publication
88846 (Protein ID in JGI)	*Physcomitrella patens 2*	Unknown	No publication
110579 (Protein ID in JGI)	*Physcomitrella patens 3*	Unknown	No publication
132698 (Protein ID in JGI)	*Physcomitrella patens 4*	Unknown	No publication
146006 (Protein ID in JGI)	*Physcomitrella patens 5*	Unknown	No publication
153672 (Protein ID in JGI)	*Physcomitrella patens 6*	Unknown	No publication
179535 (Protein ID in JGI)	*Physcomitrella patens 7*	Unknown	No publication

Supplemental references:

1. Burton, R. A., Johnson, P. E., Beckles, D. M., Fincher, G. B., Jenner, H. L., Naldrett, M. J., and Denyer, K. (2002) *Plant Physiol* **130**, 1464-1475

2. Bae, J. M., Giroux, M., and Hannah, L. (1990) *Maydica* **35**, 317-322

3. Hannah, L. C., Shaw, J. R., Giroux, M. J., Reyss, A., Prioul, J.-L., Bae, J.-M., and Lee, J.-Y. (2001) *Plant Physiol* **127**, 173-183

4. Johnson, P. E., Patron, N. J., Bottrill, A. R., Dinges, J. R., Fahy, B. F., Parker, M. L., Waite, D. N., and Denyer, K. (2003) *Plant Physiol* **131**, 684-696

5. Thorbjornsen, T., Villand, P., Kleczkowski, L. A., and Olsen, O. A. (1996) *Biochem J* **313**, 149-154

6. Ohdan, T., Francisco, P.B. Jr, Sawada, T., Hirose, T., Terao, T., Satoh, H., and Nakamura, Y. (2005) *J Exp Bot* **56,** 3229-3244

7. Akihiro. T., Mizuno. K., and Fujimura, T. (2005) *Plant Cell Physiol* **46,** 937-946

8. Sikka, V. K., Choi, S.-B., Kavakli, I. H., Sakulsingharoj, C., Gupta, S., Ito, H., and Okita, T. W. (2001) *Plant Sci* **161**, 461-468

9. Singh, S., Choi, S. B., Modi, M. K., and Okita, T. W. (2002) *Phytochemistry* **59**, 261-268

10. Vigeolas, H., Mohlmann, T., Martini, N., Neuhaus, H.E., and Geigenberger, P. (2004) *Plant Physiol* **136,** 2676-2686

11. Kim, I.-J., Park, J. Y., Lee, Y.-W., Chung, W.-I., and Lim, Y. P. (2002) *J Plant Biotechnology* **4**, 59-65

12. Burgess, D., Penton, A., Dunsmuir, P., and Dooner, H. (1997) *Plant Mol Biol* **33**, 431-444.

13. Weber, H., Heim, U., Borisjuk, L., and Wobus, U. (1995) *Planta* **195**, 352-361

14. Omoto, D., H. Ito, Y. Obana, K. Matsumoto, N. Isono, S. Hamada, T.W. Okita and H. Matsui. (2003) *J Appl Glycosci* **50**, 475-479

15. Nakata, P. A., and Okita, T. W. (1996) *Mol Gen Genet* **250**, 581-592

16. Nakata, P. A., Anderson, J. M., and Okita, T. W. (1994) *J Biol Chem* **269**, 30798-30807

17. Kim, Y. S., Lee, M. H., Shin, H. S., Lee, D. S., Son, C. B., Song, H. W., Yoo, O. J., and Liu, J. R.(1995) *Mol Cells* **5**, 605-610

18. Bae, J. M., and Liu, J. R. (1997) *Mol Gen Genet* **254**, 179-185

19. Chen, B.-Y., Janes, H. W., and Gianfagna, T. (1998) *Plant Sci* **136**, 59-67

20. Choi, S. B., Kim, K. H., Kavakli, I. H., Lee, S. K., and Okita, T. W. (2001) *Plant Cell Physiol* **42**, 146-153

21. Villand, P., Olsen, O. A., and Kleczkowski, L. A. (1993) *Plant Mol Biol* **23**, 1279-1284.
Plant J. **13**, 63-70

22. Crevillen, P., Ventriglia, T., Pinto, F., Orea, A., Merida, A., and Romero, J.M. (2005) *J Biol Chem* **280,** 8143-8149

23. Kim, I. J., Kahng, H. Y., and Chung, W. I. (1998) *Biosci Biotechnol Biochem* **62**, 550-555

24. Kim, I.-J., Noh, S.-J., Lee, B.-H., Jo, J., Kim, Y.-S., and Chung, W.-I. (2001) *Biochim Biophys Acta* **1518**, 324-328

25. Müller-Röber, B., Nast, G., and Willmitzer, L. (1995) *Plant Mol Biol* **27**, 191-197

26. Park, S. W., Kahng, H. Y., Kim, I. J., Park, J. O., and Chung, W. I. (1998) *J Plant Res* **111**, 59-63

27. Wang, S.-M., Lue, W.-L., Yu, T.-S., Long, J.-H., Wang, C.-N., Eimert, K., and Chen, J. (1998) *Plant J* **13,** 63-70

28. Ainsworth, C., Hosein, F., Tarvis, M., Weir, F., Burrell, M., Devos, K. M., and Gale, M. D. (1995) *Planta* **197**, 1-10

29. Ral,J.P., Derelle,E., Ferraz,C., Wattebled,F., Farinas,B., Corellou,F., Buleon,A., Slomianny,M.C., Delvalle,D., D'Hulst,C., Rombauts,S., Moreau,H. and Ball,S. (2004) *Plant Physiol* **136**, 3333-3340

30. Bhave, M.R., Lawrence, S., Barton, C., and Hannah, L.C. (1990) *Plant Cell* **2,** 581-588

31. Giroux, M., Smith-White, B., Gilmore, V., Hannah, L. C., and Preiss, J. (1995) *Plant Physiol* **108**, 1333-1334

32. Eimert, K., Luo, C., Dejardin, A., Villand, P., Thorbjornsen, T., and Kleczkowski, L. A. (1997) *Gene* **189**, 79-82

33. Villand, P., Aalen, R., Olsen, O. A., Luthi, E., Lonneborg, A., and Kleczkowski, L. A. (1992) *Plant Mol Biol* **19**, 381-389

34. Lacognata, U., Willmitzer, L., and Müller-Röber, B. (1995) *Mol Gen Genet* **246**, 538-548

35. Nakata, P. A., and Okita, T. W. (1995) *Plant Physiol* **108**, 361-368

36. Harn, C. H., Bae, J. M., Lee, S. S., Min, S. R., and Liu, J. R. (2000) *Plant Cell Physiol* **41**, 1235-1242

37. Park, S. W., and Chung, W. I. (1998) *Gene* **206**, 215-221.

38. Schaffer, A. A., Levin, I., Oguz, I., Petreikov, M., Cincarevsky, F., Yeselson, Y., Shen, S., Gilboa, N., and Bar, M. (2000) *Plant Sci* **152**, 135-144

39. Rook, F., Corke, F., Card, R., Munz, G., Smith, C., and Bevan, M. W. (2001) *Plant J* **26**, 421-433

40. Kavakli, I. H., Kato, C., Choi, S. B., Kim, K. H., Salamone, P. R., Ito, H., and Okita, T. W. (2002) *Planta* **215**, 430-439

41. Li, C. R., Zhang, X. B., and Hew, C. S. (2003) *Biol Plantarum* **47**, 613-615

42. Zabawinski, C., Van den Koornhuyse, N., D'Hulst, C., Schlichting, R., Giersch, C., Delrue, B., Lacroix, J. M., Preiss, J., and Ball, S. (2001) *J Bacteriol* **183**, 1069-1077

43. Charng, Y. Y., Kakefuda, G., Iglesias, A. A., Buikema, W. J., and Preiss, J. (1992) *Plant Mol Biol* **20**, 37-47

44. Kakefuda, G., Charng, Y.Y., Iglesias, A.A., McIntosh, L., and Preiss, J. (1992) *Plant Physiol.* **99**, 359-361
